# TSC1/mTOR-controlled metabolic–epigenetic cross talk underpins DC control of CD8^+^ T-cell homeostasis

**DOI:** 10.1371/journal.pbio.3000420

**Published:** 2019-08-21

**Authors:** Lei Shi, Xia Chen, Aiping Zang, Tiantian Li, Yanxiang Hu, Shixin Ma, Mengdie Lü, Huiyong Yin, Haikun Wang, Xiaoming Zhang, Bei Zhang, Qibin Leng, Jinbo Yang, Hui Xiao

**Affiliations:** 1 School of Life Sciences, Lanzhou University, Lanzhou, Gansu, China; 2 CAS Key Laboratory of Molecular Virology and Immunology, Institut Pasteur of Shanghai; CAS Center for Excellence in Molecular Cell Science; University of Chinese Academy of Sciences, Chinese Academy of Sciences, Shanghai, China; 3 Department of Immunology, Medical College of Qingdao University, Qingdao, Shandong, China; 4 Affiliated Cancer Hospital and Institute of Guangzhou Medical University, State Key Laboratory of Respiratory Diseases, Guangzhou, Guangdong, China; 5 CAS Key Laboratory of Nutrition, Metabolism and Food Safety, Shanghai Institute of Nutrition and Health, Chinese Academy of Sciences, Shanghai, China; National Cancer Institute, UNITED STATES

## Abstract

Dendritic cells (DCs) play pivotal roles in T-cell homeostasis and activation, and metabolic programing has been recently linked to DC development and function. However, the metabolic underpinnings corresponding to distinct DC functions remain largely unresolved. Here, we demonstrate a special metabolic–epigenetic coupling mechanism orchestrated by tuberous sclerosis complex subunit 1 (TSC1)-mechanistic target of rapamycin (mTOR) for homeostatic DC function. Specific ablation of *Tsc1* in the DC compartment (*Tsc1*^DC-KO^) largely preserved DC development but led to pronounced reduction in naïve and memory–phenotype cluster of differentiation (CD)8^+^ T cells, a defect fully rescued by concomitant ablation of *mTor* or regulatory associated protein of MTOR, complex 1 (*Rptor*) in DCs. Moreover, *Tsc1*^DC-KO^ mice were unable to launch efficient antigen-specific CD8^+^ T effector responses required for containing *Listeria monocytogenes* and B16 melanomas. Mechanistically, our data suggest that the steady-state DCs tend to tune down de novo fatty acid synthesis and divert acetyl-coenzyme A (acetyl-CoA) for histone acetylation, a process critically controlled by TSC1-mTOR. Correspondingly, TSC1 deficiency elevated acetyl-CoA carboxylase 1 (ACC1) expression and fatty acid synthesis, leading to impaired epigenetic imprinting on selective genes such as major histocompatibility complex (MHC)-I and interleukin (IL)-7. Remarkably, tempering ACC1 activity was able to divert cytosolic acetyl-CoA for histone acetylation and restore the gene expression program compromised by TSC1 deficiency. Taken together, our results uncover a crucial role for TSC1-mTOR in metabolic programing of the homeostatic DCs for T-cell homeostasis and implicate metabolic-coupled epigenetic imprinting as a paradigm for DC specification.

## Abstract 2

Dendritic cells (DCs) play pivotal roles in T cell homeostasis and activation, but the basis of the metabolic programming of distinct DC functions remains unclear. This study identifies a novel metabolic-epigenetic node enabling DC control of CD8 T cell homeostasis, involving mTOR-ACC1 as a rheostat that balances fatty-acid synthesis and histone acetylation.

## Introduction

Dendritic cells (DCs) are specialized sentinel cells of immune system responsible for the initiation and coordination of innate and adaptive immunity [1,2]. It has become increasingly appreciated that DCs encompass phenotypic and functional heterogeneous subsets, which can be further diversified in different anatomic locations, such as the spleen, lymph nodes (LNs), and peripheral tissues [3]. Whereas plasmacytoid DCs (pDCs) are professional interferon (IFN)-I producers required for the imminent control of viral infections, the classical DCs (cDCs) comprising cDC1 and cDC2 are specialized in antigen presentation and dedicated to T-cell priming and effector differentiation [4,5]. Recent advancement indicates that DCs not only are central to T-cell activation but also hold the keys for immune tolerance and homeostasis [2,4]. Although the principles governing DC control of T-cell activation have been established, the mechanisms underlying DC control of T-cell homeostasis remain underappreciated. Given the broad association of corruptions in immune homeostasis with human diseases, particularly autoimmune diseases and cancers, it is of great interest to understand how DCs regulate immune homeostasis.

T lymphocytes generated in the thymus can be maintained as naïve cells in the periphery for a relatively long period [6]. During the naïve state, T cells are believed to stay in quiescence and, thus, barely divide [7,8]. However, quiescent cluster of differentiation (CD)8^+^ T cells must engage self-peptide/major histocompatibility complex (MHC)-I complexes for tonic T-cell receptor (TCR) signaling to survive in the periphery [9]. As MHC class I molecules are ubiquitously expressed, both DCs and non-DCs have been shown to be able to present autoantigens to the naïve CD8^+^ T cells [10,11]. In addition, CD8^+^ T cells also depend on interleukin (IL)-7 for their periphery maintenance, and those unable to respond to IL-7 become rapidly lost in the periphery [12,13]. Although stroma cells such as fibroblast reticular cells (FRCs) are regarded as the major cellular source for IL-7, evidence implicating DCs as a possible IL-7 provider has also emerged [12,14,15]. In the steady state, a proportion of CD8^+^ T cells spontaneously convert into so-called memory–phenotype T cells with CD62L^lo^ and CD44^hi^ surface markers [8]. How these memory–phenotype CD8^+^ T cells arise remains unclear, but their maintenance and survival seem contingent on cytokines IL-7 and IL-15 [6,8]. Notably, the numbers of naïve CD8^+^ T cells must be kept constant and steady throughout their life, and central to this precision control is the competition for the limited IL-7 and self-peptide/MHC-I resources among CD8^+^ T cells [9]. The molecular mechanisms underlying the stringent control of IL-7 production and MHC class I–restricted antigen presentation are poorly understood. Because disruption of T-cell quiescence can lead to autoimmunity and lymphopenia, the balance of being quiescent while surviving is critically maintained by both T cell–intrinsic and–extrinsic signals.

With prominent expression of a diversity of pattern-recognition receptors (PRRs), DCs are specialized in recognition of pathogen-associated molecule patterns (PAMPs) and danger-associated molecule patterns (DAMPs) associated with pathogen infection and tissue injury, respectively [2]. Engagement of PRRs such as Toll-like receptors (TLRs) and C-type lectin receptors (CLRs) leads to DC activation, characterized with up-regulated capacities in antigen presentation, migration, and cytokine production. The emerging view suggests metabolic reprograming as an integral component of DC activation [16,17]. TLR-activated DCs rapidly elevate glucose uptake and oxidative glycolysis, leading to marked increase in de novo fatty acid synthesis [18]. Interestingly, this metabolic program features lipid production and appears to fit into the demand for endoplasmic reticulum (ER) and Golgi expansion, allowing activated DCs to produce a myriad of cytokines and chemokines. Consistent with this theme, mechanistic target of rapamycin (mTOR)-regulated fatty acid oxidation (FAO) has been implicated in DC regulation of allergic T helper 2 (Th2) response [19]. Therefore, different metabolic programs must incorporate into distinct DC subsets at various anatomic locations to accommodate respective functions [20]. In this regard, a special metabolic program featured a high level of oxidative phosphorylation (OXPHOS) and, orchestrated by mammalian sterile-20-like 1/2 (Mst1/2), was only associated with cDC1 but not cDC2 subset [21]. It is noteworthy that metabolic reprograming has been linked to epigenetic regulation of gene expression, largely because of their profound impact on the cofactors of epigenetic enzymes, such as S-adenosylmethionine (SAM) and acetyl-coenzyme A (acetyl-CoA), which are used by histone methyltransferase and acetyltransferase to shape the chromatin landscapes [22–25]. Indeed, macrophages stimulated with IL-4 exhibited pronounced FAO and up-regulation of ATP-citrate lyase (Acly), leading to increased acetyl-CoA production and histone acetylation for selective alternatively activated macrophage (M2 macrophage)-specific gene expression [26]. However, whether and how DC-specific metabolic programs might intersect with diverse epigenetic mechanisms awaits investigation.

Recent development has placed mTOR at the center of immune regulation, highlighting its multifaceted functions in a wide array of immune cells. mTOR constitutes two multicomponent complexes, mTOR complex 1 (mTORC1) and mTOR complex 2 (mTORC2), and through these complexes shape the cellular metabolic programs to meet the specific bioenergetic and biosynthetic needs of immune cells [20]. Whereas mTORC1 has been widely linked to anabolic metabolism through the downstream effectors hypoxia-inducible factor-1α (HIF-1α) and cellular myelocytomatosis (c-Myc), mTORC2 can also participate in the regulation of glycolysis in part through up-regulating c-Myc expression [17,20]. It is becoming increasingly evident that mTOR activity varies in immune cells at distinct activation stages or different anatomic locations, partly attributing to the upstream negative regulators such as tuberous sclerosis complex subunit 1/2 (TSC1/2), AMP-activated protein kinase (AMPK), and phosphatase and tensin homolog (PTEN) [27]. TSC1/TSC2 heterocomplexes are responsible for receiving and integrating signals from cytokines, growth factors, and PRRs, thereby executing stringent control on mTORC1. Not surprisingly, precision control of mTOR activation has been implicated in DC survival and inflammation [28–30]. In this study, we established a crucial role for TSC1-mTOR in the steady-state DCs and unraveled a unique metabolic–epigenetic program underpinning DC-mediated CD8^+^ T-cell survival. Importantly, our data implicate the existence of a novel TSC1-mTOR–acetyl-CoA carboxylase 1 (ACC1) axis operating at the interface of fatty acid synthesis and histone acetylation, thus providing important insight into the understanding of metabolic–epigenetic coupling mechanisms. It is also noteworthy that this study serves as a unique paradigm supporting DC’s involvement in the peripheral CD8^+^ T-cell maintenance.

## Results

### DC expression of TSC1 is pivotal for CD8^+^ T-cell development and maintenance

**Fig 1:**
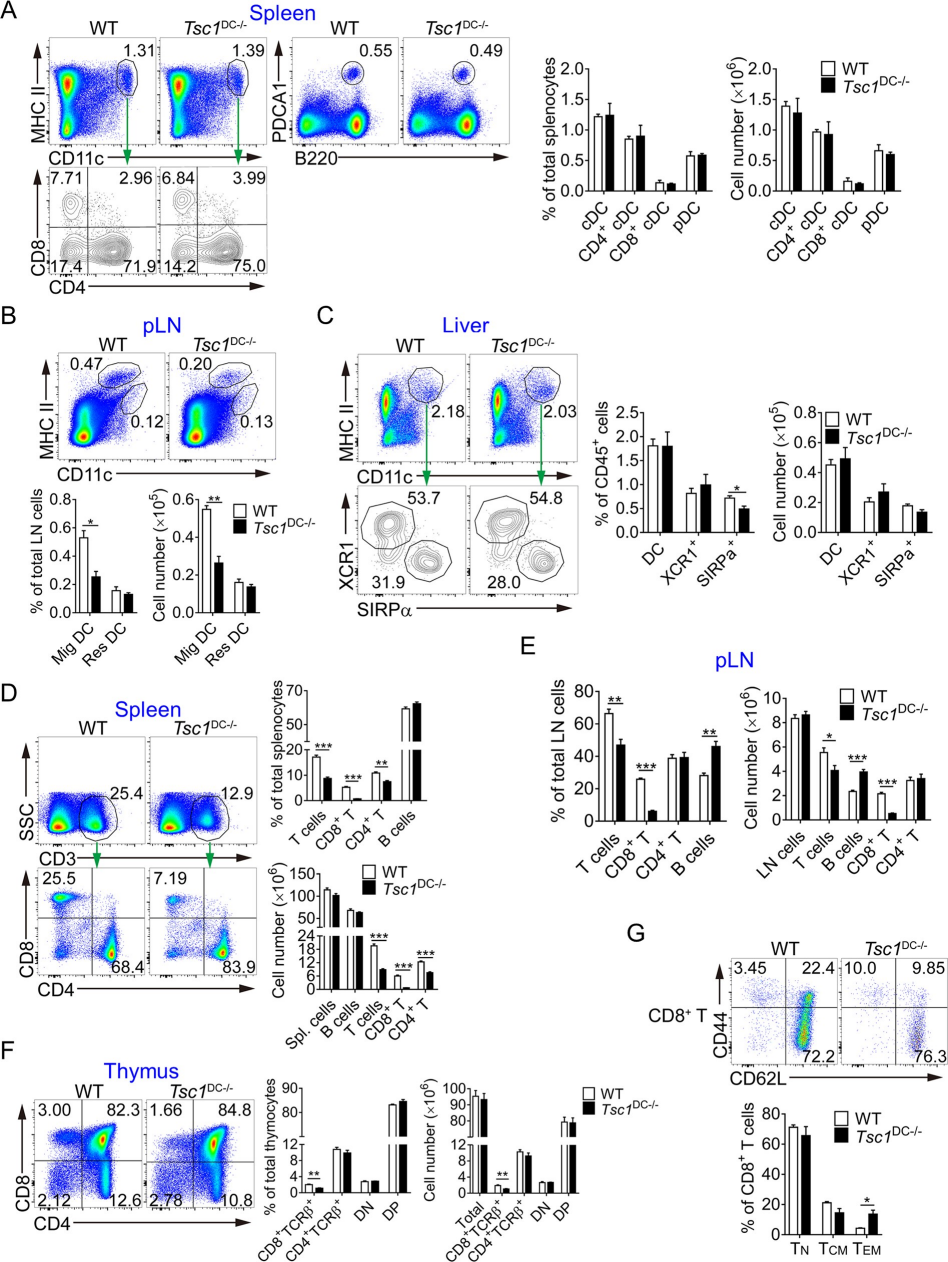
Analyses of DC and T-cell populations in TSC1^DC-KO^ mice. (A and B) The percentages and numbers of cDCs and pDCs in the spleen (A) and the resident and mig DCs in the pLNs (B) of WT and TSC1^DC-KO^ mice ([A] *n* = 4; [B] *n* = 3) were analyzed by flow cytometry, and the total cell numbers were counted by a hemocytometer under a microscope. These experiments were repeated three times with similar results. (C) The percentages and numbers of cDCs (CD11c^+^MHC-II^+^, pregated as F4/80^−^CD64^−^) and XCR1^+^ and SIRPα^+^ DC subsets in the livers of WT and TSC1^DC-KO^ mice (*n* = 6). This experiment was conducted twice, and similar results were obtained. (D and E) The percentages and numbers of T and B cells of Spls. (D) or pLNs (E) from WT and TSC1^DC-KO^ mice (*n* = 4) were analyzed by flow cytometry. These experiments were conducted three times, and similar results were obtained. (F) The percentages and numbers of different T-cell populations in the thymuses of WT and TSC1^DC-KO^ mice (*n* = 4) were analyzed by flow cytometry. This experiment was conducted three times with similar results. (G) The T_N_ and memory–phenotype CD8^+^ T cells of WT and TSC1^DC-KO^ Spls. (*n* = 4) were analyzed by flow cytometry, and the percentages were calculated. This experiment was repeated twice with similar results. All the data are presented as means ± SEM (*p < 0.05, **p < 0.01, ***p < 0.01; analyzed by Student’s *t* test). Underlying data are available in S1 Data. CD, cluster of differentiation; cDC, classical DC; DC, dendritic cell; DN, double negative; DP, double positive; LN, lymph node; MHC, major histocompatibility complex; Mig DC, migratory DC; pDC, plasmacytoid DC; PDCA1, pDC Ag-1; pLN, peripheral lymph node; Res DC, resident DC; SIRPα, signal regulatory protein α; Spl., spleen; SSC, side scatter; TCR, T-cell receptor; T_CM_, central memory T cell; T_EM_, effector memory T cell; T_N_, naïve T cell; Tsc1, tuberous sclerosis complex subunit 1; TSC1^DC-KO^, specific ablation of *Tsc1* in the DC compartment; WT, wild-type; XCR1, chemokine (C motif) receptor 1.

To comprehensively understand the role of TSC1-mTOR and corresponding metabolic programs in DC function, we generated *Tsc1* floxed:Cd11c-Cre (cyclization recombination enzyme) mouse model to achieve specific ablation of *Tsc1* in the DC compartment (referred to as TSC1^DC-KO^ hereafter). TSC1^DC-KO^ mice were born at the mendelian ratio and appeared grossly normal up to 1 year of age. The examination of *Tsc1* expression by quantitative real-time PCR and immunoblotting revealed drastic reduction of TSC1 expression in splenic CD11c^+^ DCs (**S1A** and **S1B Fig**), indicating efficient ablation of *Tsc1* alleles in the DC compartment. As expected, unaltered TSC1 expression was associated with macrophages (CD11b^+^), natural killer cells (NKs) (NK1.1^+^), B cells (B220^+^), and CD8^+^ T cells isolated from TSC1^DC-KO^ spleens (**S1A** and **S1B Fig**). Intriguingly, unexpected reduction in TSC1 expression was observed in the CD4^+^ T cells from TSC1^DC-KO^ spleens, particularly at the mRNA level (**S1A** and **S1B Fig**). Next, we examined how TSC1 deficiency might influence DC development and differentiation. Flow cytometry analyses revealed a largely normal presence of various DC subsets in the major lymphoid organs of TSC1^DC-KO^ mice, including the thymus, spleen, and LNs (**Fig 1A** and **1B** and **S1C Fig**). Specifically, the percentages and numbers of CD8α^+^ DCs (cDC1), CD11b^+^ or CD4^+^ DCs (cDC2), and pDC Ag-1 (PDCA1)^+^/B220^+^ pDCs were comparable in the thymuses and spleens of wild-type and TSC1^DC-KO^ mice (**Fig 1A** and **S1C Fig**), indicating a dispensable role for TSC1 in DC lineage commitment and differentiation. Moreover, the LNs from TSC1^DC-KO^ mice constituted unaltered resident DCs (CD11c^hi^/MHC-II^int^) but fewer migratory DCs (CD11c^int^/MHC-II^hi^) as compared with the wild-type LNs (**Fig 1B**). Notably, the migratory DCs from TSC1^DC-KO^ LNs exhibited normal expression of chemokine (C-C motif) receptor 7 (CCR7) (**S1D Fig**). Furthermore, the percentages and numbers of chemokine (C motif) receptor 1 (XCR1)^+^ or CD103^+^ DCs (cDC1) and CD11b^+^ or signal regulatory protein α (SIRPα)^+^ DCs (cDC2) were also similarly present in the livers and kidneys of TSC1^DC-KO^ mice (**Fig 1C** and **S1E Fig**). Hence, TSC1 deficiency did not seem to affect DC development and maintenance in the lymphoid organs.

Subsequently, we investigated whether TSC1 deficiency might affect DC function, with the focus on adaptive immune cells. The percentages and numbers of B lymphocytes were largely normal in the TSC1^DC-KO^ secondary lymphoid organs, with the exception of peripheral LNs (pLNs), where B cells increased (**Fig 1D** and **1E**). In contrast, marked reduction in CD3^+^ T lymphocytes was detected in TSC1^DC-KO^ mice (**Fig 1D** and **1E** and **S1F** and **S1G Fig**). Among the CD3^+^ T-cell populations, CD8^+^ T cells showed a substantial decrease in percentages and numbers crossing various lymphoid organs, whereas CD4^+^ T cells were normally present in the LNs but slightly diminished in the spleens of TSC1^DC-KO^ mice (**Fig 1D** and **1E** and **S1F** and **S1G Fig**). These results thus revealed an unexpected association of DC-restricted TSC1 expression with peripheral CD8^+^ T-cell homeostasis.

As naïve T cells developed in the thymus continuously replenish peripheral tissues, we then examined whether T-cell development was affected in TSC1^DC-KO^ mice. The presence of both double-negative and double-positive T cells was unimpaired in the thymuses of TSC1^DC-KO^ mice, suggestive of normal positive- and negative-selection processes. However, whereas CD4^+^ TCRβ^+^ T cells were generated at the expected percentage, the CD8^+^ TCRβ^+^ T cells were modestly underrepresented (about 2-fold reduction) in the TSC1^DC-KO^ thymuses (**Fig 1F**), implicating compromised CD8^+^ T-cell development by TSC1 DC deficiency. In the periphery, the majority of the CD8^+^ T cells were kept at the naïve state, whereas a portion of them also spontaneously converted to memory–phenotype T cells, including central memory (CD62L^hi^CD44^hi^) and effector memory (CD62L^lo^CD44^hi^) T cells. In TSC1^DC-KO^ mice, the ratios of naïve and central memory subsets were slightly decreased, whereas the effector memory T-cell percentages increased within the CD8 and CD4 T-cell populations (**Fig 1G** and **S1H Fig**). Collectively, these data demonstrate a crucial role for TSC1 in DC function to instruct CD8^+^ T-cell development and maintenance.

### DC mTORC1 activation controlled by TSC1 is crucial for CD8^+^ T-cell survival

**Fig 2:**
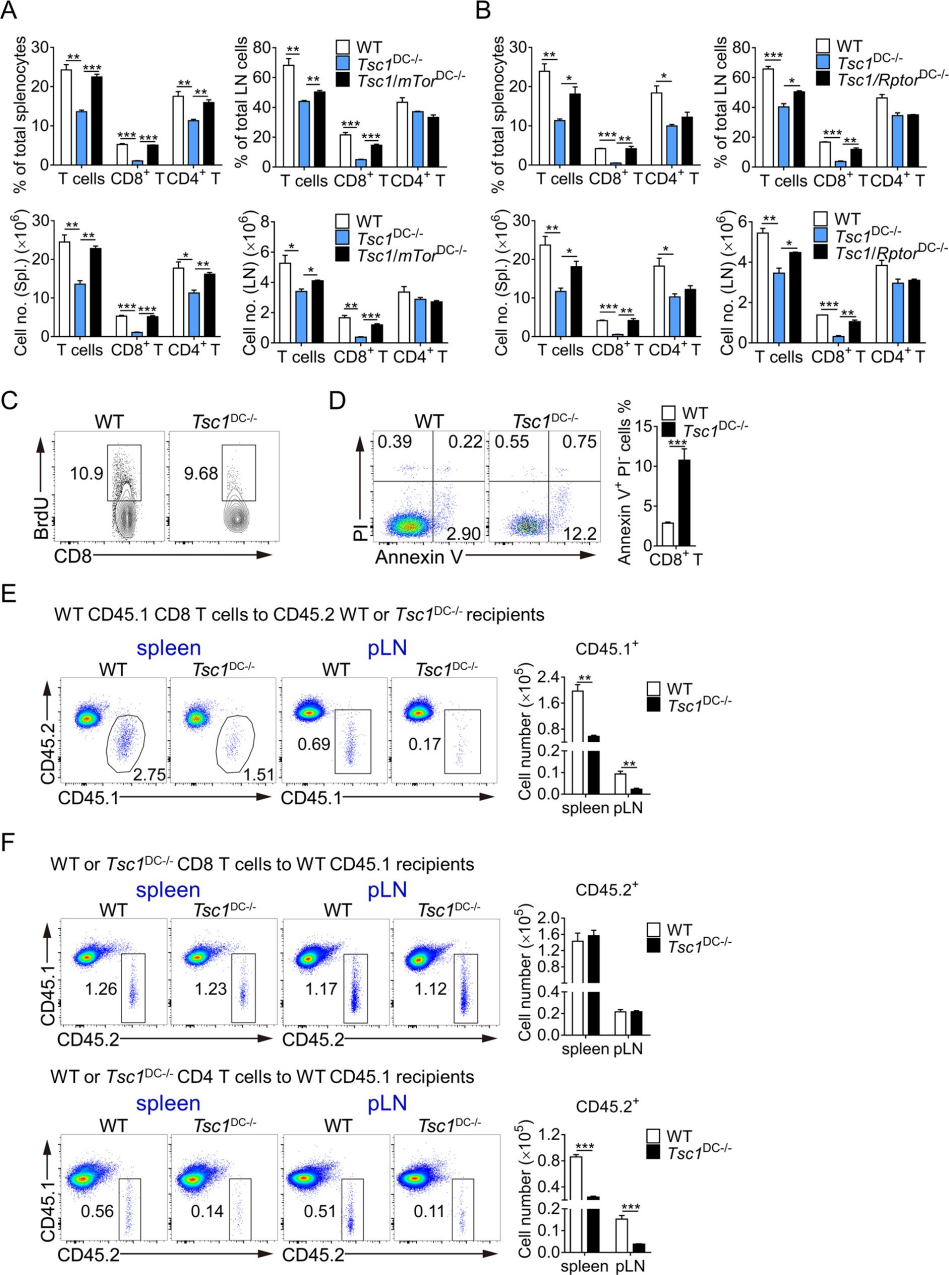
mTORC1 in the DC compartment controls CD8^+^ T-cell survival. (A and B) The percentages and numbers of total T cells (CD3^+^) and T-cell subsets (CD8^+^ and CD4^+^ T cells) of the Spls. and pLNs from WT, TSC1^DC-KO^, and TSC1/mTOR^DC-DKO^ mice (*n* = 3) (A) or TSC1/Raptor^DC-DKO^ mice (*n* = 3) (B) were analyzed by flow cytometry, and the cell percentages were calculated accordingly. These experiments were repeated twice with similar results. The data are presented as means ± SEM (*p < 0.05, **p < 0.01, and ***p < 0.001; analyzed by Student’s *t* test). (C) The 6–8-week-old WT and TSC1^DC-KO^ littermates (*n* = 3) were i.p. injected with 1 mg BrdU per mouse and then fed with 0.8 mg/ml BrdU containing water for 7 days. The splenocytes were isolated and stained for BrdU. The percentages of BrdU^+^ CD8^+^ T cells were shown. This experiment was conducted twice, and similar results were obtained. (D) Spleens from WT and TSC1^DC-KO^ mice (*n* = 6) were isolated and immediately stained with annexin V and PI; after cell surface marker staining, early apoptotic CD8^+^ T cells (annexin V^+^PI^−^) were calculated. This experiment was repeated twice with similar results. The data are presented as means ± SEM (***p < 0.001, analyzed by Student’s *t* test). (E) WT CD45.1 donor CD8^+^ T cells from Spl. (5 × 10^6^ CD8^+^ T cells) were transferred into WT and TSC1^DC-KO^ recipient mice (*n* = 3), and the percentages of transferred CD45.1 CD8^+^ T cells in the Spls. and pLNs were analyzed by flow cytometry after 2 weeks. This experiment was repeated twice with similar results. The data are presented as means ± SEM (**p < 0.01, analyzed by Student’s *t* test). (F) In total, 5 × 10^6^ CD45.2 donor CD8^+^ T cells or CD4^+^ T cells from WT and TSC1^DC-KO^ Spls. were transferred into CD45.1 recipient mice (*n* = 4), and the percentages of transferred CD45.2 cells in the Spls. and pLNs were analyzed by flow cytometry after 2 weeks. This experiment was repeated twice with similar results. The data are presented as means ± SEM (***p < 0.001, analyzed by Student’s *t* test). Underlying data are available in S1 Data. BrdU, bromodeoxyuridine; CD, cluster of differentiation; DC, dendritic cell; i.p., intraperitoneally; LN, lymph node; mTOR, mechanistic target of rapamycin; mTORC1, mTOR complex 1; PI, propidium iodide; pLN, peripheral LN; Rptor, regulatory associated protein of MTORc1; Spl., spleen; Tsc1, tuberous sclerosis complex subunit 1; TSC1^DC-KO^, specific ablation of *Tsc1* in the DC compartment; WT, wild-type.

Considering the well-established link of TSC1 to mTORC1, we then tested whether hyperactive mTORC1 might have contributed to DC dysfunction in TSC1^DC-KO^ mice. To this end, we crossed *mTor* and regulatory associated protein of MTORc1 (*Rptor*) floxed mouse strains onto *Tsc1* floxed:Cd11c-Cre mice, thereby generating TSC1/mTOR^DC-DKO^ and TSC1/Raptor^DC-DKO^ mice, respectively. Remarkably, concomitant ablation of mTOR and TSC1 in the DC compartment resulted in full restoration of T lymphocytes in the spleens and LNs of TSC1/mTOR^DC-DKO^ mice (**Fig 2A** and **S2A Fig**). Moreover, TSC1/Raptor^DC-DKO^ mice showed completely normalized CD8^+^ T-cell populations, but still with CD4^+^ T-cell population impaired in the spleen (**Fig 2B** and **S2B Fig**), underscoring mTORC1 as the chief target for TSC1 in this process. Collectively, these data establish a crucial role for TSC1-mTORC1 in DC-mediated CD8^+^ T-cell homeostasis.

A number of checkpoints, such as development, proliferation/survival, and quiescence, have been implicated in T-cell homeostasis. Because CD8^+^ T cells were only slightly decreased (approximately 40% reduction) in the thymus of TSC1^DC-KO^ mice (**Fig 1F**), the developmental process alone cannot account for nearly 90% of CD8^+^ cell reduction in the spleen and LNs (**Fig 1D** and **1E**). Hence, we reasoned that processes like cell proliferation and survival might be affected in TSC1^DC-KO^ mice. Upon administering bromodeoxyuridine (BrdU) via intraperitoneal injection followed by continuous feeding in drinking water for 7 days [31], we observed very few BrdU-positive CD8^+^ T cells in the spleen, and the percentages of BrdU^+^ CD8^+^ T cells were comparable in both wild-type and TSC1^DC-KO^ mice (**Fig 2C**). Conversely, CD8^+^ T cells isolated from TSC1^DC-KO^ spleens displayed nearly 5-fold more annexin V–positive apoptotic cells (**Fig 2D**), indicative of impaired CD8^+^ T-cell survival. Meanwhile, increased apoptosis was also associated with TSC1^DC-KO^ splenic CD4^+^ T cells (**S2C Fig**), corroborating the decreased CD4^+^ T-cell population in the TSC1^DC-KO^ spleen (**Fig 1D**).

Subsequently, we conducted a series of reciprocal adoptive transfer experiments to ascertain the role of DCs in CD8^+^ T-cell defects described above. At first, CD45.1 wild-type CD8^+^ T cells were transferred into CD45.2 wild-type or TSC1^DC-KO^ recipients and traced by flow cytometry after 2 weeks. Notably, much fewer CD45.1 donor CD8^+^ T cells were recovered from the TSC1^DC-KO^ recipients than wild-type recipients (**Fig 2E**). In contrast, when CD45.2 CD8^+^ T cells from wild-type or TSC1^DC-KO^ spleens were transferred into CD45.1 wild-type recipients, both types of CD45.2 donor CD8^+^ T cells were similarly recovered from the wild-type recipients (**Fig 2F**). Nevertheless, CD45.2 CD4^+^ T donor cells from TSC1^DC-KO^ spleens were recovered with diminished numbers as compared with their counterparts from wild-type spleens (**Fig 2F**). These data collectively ruled out any possible intrinsic defect associated with the CD8^+^ T cells; however, the CD4^+^ T cells from TSC1^DC-KO^ spleen might be intrinsically compromised, which is consistent with subset CD4^+^ T-cell reduction observed in the TSC1^DC-KO^ spleens (**Fig 1D**). As CD11c expression can be associated with myeloid cell lineages other than DCs, we further examined whether the observed CD8^+^ T-cell defect could be attributable to macrophages or neutrophils. However, the CD8^+^ T-cell populations were completely normal when TSC1 was ablated in the macrophage/neutrophil compartments via lysozyme 2 (*Lyz2*)-Cre (**S2D Fig**). Together, these data demonstrate that TSC1 expression in DCs is critically involved in CD8^+^ T-cell development and maintenance.

### DC TSC1 expression is crucial for in vivo CD8^+^ T-cell activation

**Fig 3:**
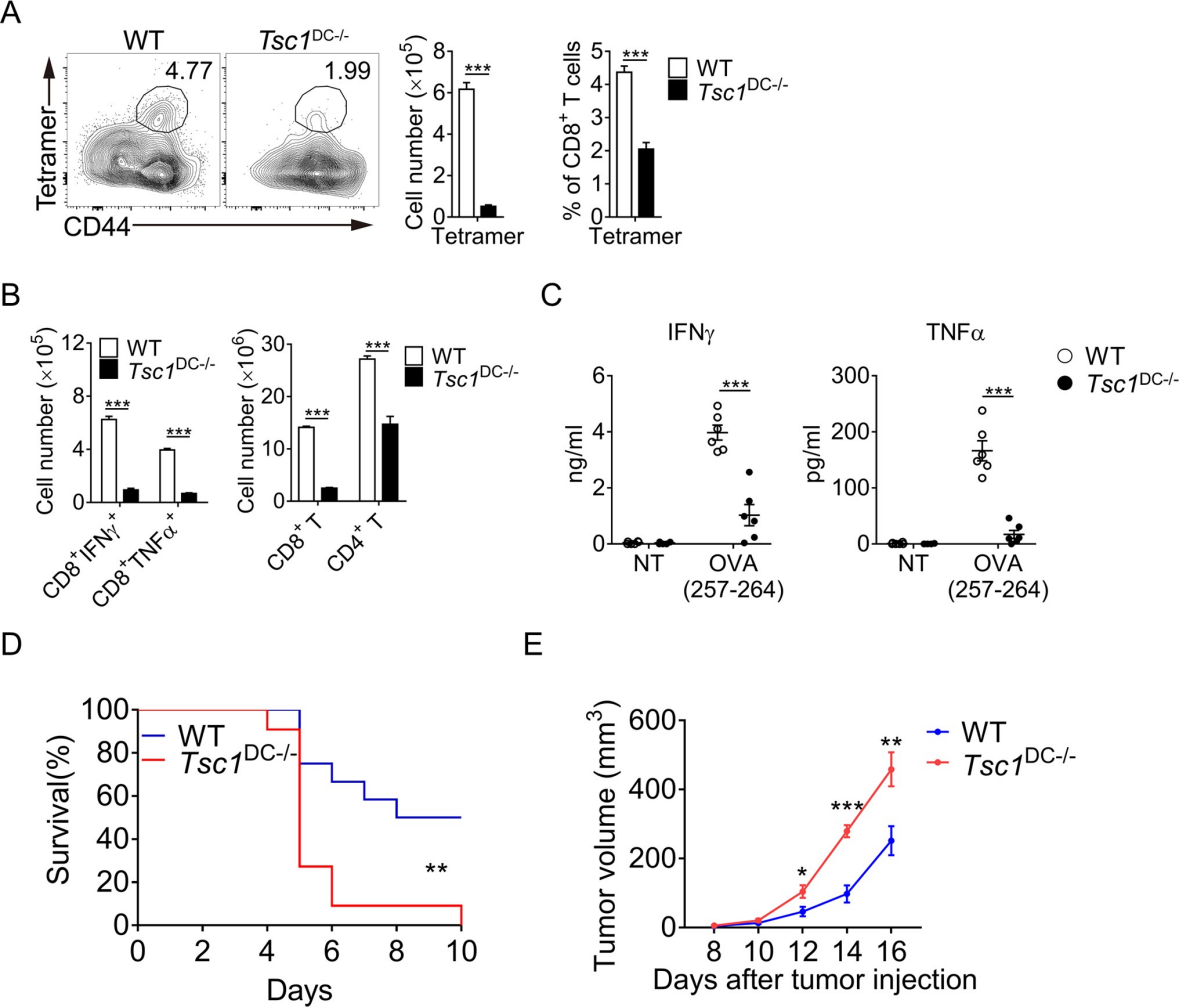
TSC1^DC-KO^ mice had impaired CD8^+^ T-cell responses. (A) The 6–8-week-old WT and TSC1^DC-KO^ littermates (*n* = 4) were i.v. infected with 10^4^ CFU of L.M.-OVA, the percentage of the OVA-specific CD8^+^ T cells were analyzed by flow cytometry after 7 days, and the numbers of tetramer-positive CD8^+^ T cells in the spleens were calculated. This experiment was repeated three times, and similar results were obtained. The data are shown as means ± SEM (***p < 0.001, analyzed by Student’s *t* test). (B) In total, 5 × 10^6^ splenocytes from L.M.-OVA-infected mice were seeded onto 24-well plates and restimulated with 10 ng/ml of OVA_257-264_ peptides for 5 hours in the presence of brefeldin A. The percentages of IFNγ- and TNF-producing CD8^+^ T cells were analyzed by intracellular staining, and the numbers of IFNγ- and TNF-producing CD8^+^ T cells as well as the total CD8^+^ and CD4^+^ T cells were counted. This experiment was repeated three times, and similar results were obtained. The data are shown as means ± SEM (***p < 0.001; analyzed by Student’s *t* test). (C) The splenocytes from infected mice were seeded into 96-well plates (10^6^ cells per well) and restimulated with 10 ng/ml OVA_257-264_ for 48 hours. Secreted IFNγ and TNFα in the supernatant were quantified by ELISA. This experiment was conducted twice with similar results. The data are shown as means ± SEM (***p < 0.001, analyzed by Student’s *t* test). (D) The 6–8-week-old WT and TSC1^DC-KO^ littermates (*n* = 12) were i.v. infected with 5 × 10^5^ CFU of L.M.-OVA and monitored every day for mortality. This experiment was repeated once with similar results. Log-rank (Mantel–Cox) test was used for statistical analysis (**p < 0.01). (E) WT and TSC1^DC-KO^ littermates (*n* = 6) were injected s.c. with 5 × 10^5^ B16-OVA melanoma cells, and tumor size was measured every 2 days. This experiment was repeated once with similar results. The data are shown as means ± SEM (*p < 0.05, **p < 0.01, ***p < 0.001, analyzed by Student’s *t* test). Underlying data are available in S1 Data. CD, cluster of differentiation; CFU, colony-forming unit; DC, dendritic cell; IFN, interferon; i.v., intravenously; L.M., *L*. *monocytogenes*; NT, untreated; OVA, ovalbumin; s.c., subcutaneously; TNF, tumor necrosis factor; Tsc1, tuberous sclerosis complex subunit 1; TSC1^DC-KO^, specific ablation of *Tsc1* in the DC compartment; WT, wild-type.

Given DC’s crucial roles in triggering cytolytic T-cell response, we then investigated how TSC1-deficiency in DCs might affect CD8^+^ T-cell response against intracellular pathogens and malignancy. Bacteria *L*. *monocytogenes* (L.M.) is a kind of gram-positive intracellular pathogen whose clearance requires potent CD8^+^ T-cell response. Upon intravenous infection with L.M.-ovalbumin (OVA), a recombinant strain expressing chicken OVA for 7 days, antigen-specific T-cell responses were analyzed in both wild-type and TSC1^DC-KO^ mice. Notably, tetramer staining revealed a marked decrease in OVA-specific CD8^+^ T-cell induction in TSC1^DC-KO^ mice (**Fig 3A**). Moreover, TSC1^DC-KO^ mice also had much fewer activated CD8^+^ T cells compared with wild-type mice, exhibiting pronounced reduction in both CD44^+^ killer cell lectin-like receptor subfamily G, member 1 (KLRG1)^+^ effector cells and CD44^+^KLRG1^−^ memory cells (**S3A Fig**). Among the activated T cells, the numbers of IFNγ- and tumor necrosis factor (TNF)-producing CD8^+^ T cells were also substantially diminished in L.M.-infected TSC1^DC-KO^ mice (**Fig 3B** and **S3B Fig**). Remarkably, the induction of tetramer^+^ and IFNγ^+^ CD8^+^ T cells was fully restored in the TSC1/mTOR^DC-DKO^ mice (**S3C Fig**), indicating a crucial role for TSC1-mTOR in DC-mediated CD8^+^ T-cell activation. Furthermore, upon stimulation of splenocytes isolated from L.M.-infected mice with OVA_257-264_ peptides ex vivo, it was evident that the OVA-specific CD8^+^ T cells from TSC1^DC-KO^ mice secreted very little IFNγ and TNF, indicative of compromised ability to eradicate infected bacteria (**Fig 3C**). Consequently, TSC1^DC-KO^ mice became extremely vulnerable to L.M. infection and suffered 100% mortality by 10 days of infection (**Fig 3D**).

Consistent with the crucial role for CD8^+^ T response in the elimination of malignant cancer, TSC1^DC-KO^ mice were unable to efficiently control the subcutaneously transplanted B16 melanomas. Consequently, massive tumor growth and expansion were detected in the TSC1^DC-KO^ mice but not in the TSC1/mTOR^DC-DKO^ mice (**Fig 3E** and **S3D Fig**). These data collectively indicate that TSC1-mTORC1 in DCs is instrumental for the induction of CD8^+^ T-cell responses to bacterial infection and tumor growth.

Notably, upon activation, TSC1-deficient DCs demonstrated normal induction of cytokines (TNF, IL-6, IL-12, and IL-10) and chemokines (chemokine [C-X-C motif] ligand [CXCL]-1 and CXCL-10) (**S4A Fig**). Furthermore, the expression of costimulatory molecules (CD80/CD86 and CD40) appeared slightly increased in the TSC1-deficient DCs, whereas MHC-I expression decreased (**S4B Fig**). Additionally, the CD8^+^ T cells from TSC1^DC-KO^ mice showed increased expression of chemokine (C-X-C motif) receptor 4 (CXCR4) but unaltered expression on other trafficking receptors such as CCR7 and CD62L (**S4C Fig**). Concomitantly, the CD4^+^ T cells also expressed normal CCR7, CD62L, and IL-2, but they expressed more CXCR4 and IL-7Rα in TSC1^DC-KO^ mice (**S4C–S4E Fig**). These data are consistent with the observation that the percentages of activated CD8^+^ T cells, particularly the IFNγ- and TNF-producing CD8^+^ T cells, were comparable in both wild-type and TSC1^DC-KO^ mice (**S3B Fig**). Hence, it seems that the TSC1-deficient DCs are still capable of priming CD8 T cells, and the CD8^+^ T cells do not have overt defects in trafficking or receiving signals from CD4^+^ T cells either. Conceivably, the drastic reduction in the number of activated CD8^+^ T cells in TSC1^DC-KO^ mice may be largely due to its defective homeostasis.

### TSC1 controls a transcriptional program involved in DC–T interaction

**Fig 4:**
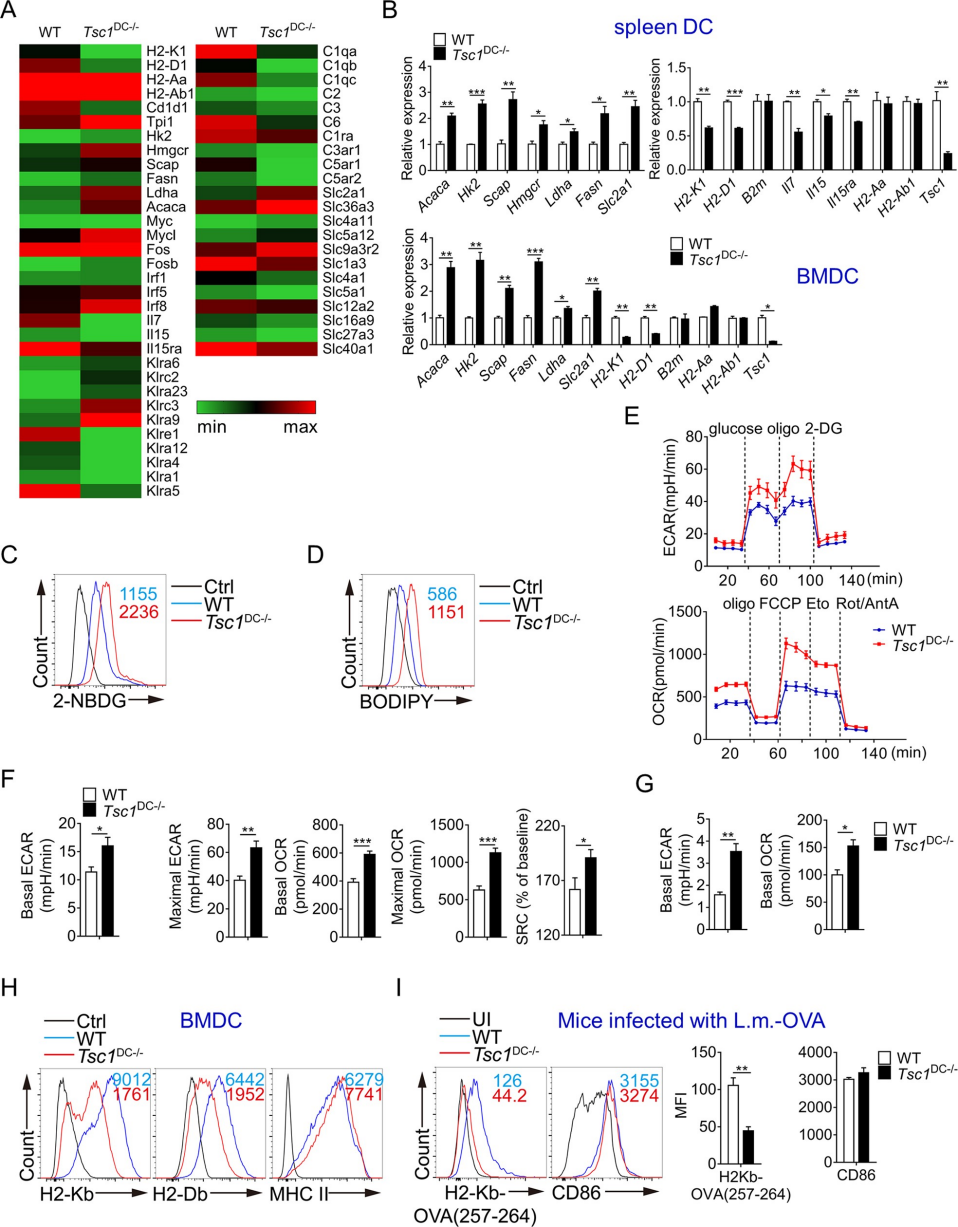
Elevated glycolysis and fatty acid synthesis in TSC1^DC-KO^ mice. (A) Genome-wide RNA-seq analysis on spleen DCs isolated from 6–8-week-old WT and TSC1^DC-KO^ littermates; the heat map shows differentially expressed gene sets, and the original data deposited in the Sequence Read Archive of NCBI database are accessible via accession No. PRJNA516783. (B) Real-time PCR analyses of gene expression in spleen DCs and BMDCs from 6–8-week-old WT and TSC1^DC-KO^ littermates. These experiments were repeated twice with similar results. The data are shown as means ± SEM (*p < 0.05, **p < 0.01, ***p < 0.001; analyzed by Student’s *t* test). (C and D) BMDCs were incubated with 50 μM 2-NBDG for 15 minutes (C) or 5 μg/ml BODIPY for 30 minutes (D) at 37°C, and the fluorescent intensity was measured by flow cytometry. These experiments were repeated once with similar results. (E and F) Real-time changes in ECAR and OCR were analyzed in WT and TSC1^DC-KO^ BMDCs by a Seahorse analyzer following sequential treatment with 10 mM glucose, 1 μM oligo, and 100 mM 2-DG or with 1 μM oligo, 1.5 μM FCCP, 200 μM Eto, and 100 nM Rot plus 1 μM AntA (Rot/AntA). Basal ECAR, maximal ECAR (after treatment with oligo), basal OCR, maximal OCR (after treatment with FCCP), and SRC were calculated accordingly (F). These experiments were repeated twice with similar results. The data are shown as means ± SEM (*p < 0.05, **p < 0.01, ***p < 0.001; analyzed by Student’s *t* test). (G) The basal ECAR and OCR of splenic DCs from WT and TSC1^DC-KO^ mice were analyzed by a Seahorse analyzer, and this experiment was conducted twice with similar results. The data are presented as means ± SEM (*p < 0.05, **p < 0.01; analyzed by Student’s *t* test). (H) The cell surface MHC-I and MHC-II expression in WT and TSC1^DC-KO^ BMDCs was analyzed by flow cytometry. This experiment was repeated once with similar results. (I) The 6–8-week-old WT and TSC1^DC-KO^ littermates (*n* = 3) were i.v. infected with 10^5^ CFU of L.M.-OVA, and the expression levels of cell surface H2-K^b^/OVA_257-264_ complexes and CD86 in splenic DCs (gated on CD11c^+^MHC-II^+^) were analyzed by flow cytometry after 3 days. This experiment was repeated once with similar results. MFI are presented as means ± SEM (**p < 0.01, analyzed by Student’s *t* test). Underlying data are available in S1 Data. 2-DG, 2-deoxy-D-glucose; 2-NBDG, 2-deoxy-2-[(7-nitro-2,1,3-benzoxadiazol-4-yl)amino]-D-glucose; Acaca, acetyl-coenzyme A carboxylase alpha; AntA, antimycin A; B2m, beta-2 microglobulin; BMDC, bone marrow–derived DC; BODIPY, boron-dipyrromethene; CD, cluster of differentiation; CFU, colony-forming unit; Ctrl, control; DC, dendritic cell; ECAR, extracellular acidification rate; Eto, etomoxir; Fasn, fatty acid synthase; FCCP, fluoro-carbonyl cyanide phenylhydrazone; H2-Aa, histocompatibility 2, class II antigen A, alpha; H2-Ab1, histocompatibility 2, class II antigen A, beta 1; H2-Db, histocompatibility 2, D region locus 1; H2-Kb, histocompatibility 2, K1, K region; Hk2, hexokinase 2; Hmgcr, 3-hydroxy-3-methylglutaryl-coenzyme A reductase; Il, interleukin; Ldha, lactate dehydrogenase A; L.M., *L*. *monocytogenes*; MFI, mean fluorescence intensity; MHC, major histocompatibility complex; NCBI, National Center for Biotechnology Information; OCR, oxygen consumption rate; oligo, oligomycin; OVA, ovalbumin; RNA-seq, RNA sequencing; Rot, rotenone; Scap, sterol regulatory element binding transcription factor chaperone; Slc2a1, solute carrier family 2 member 1; SRC, spare respiratory capacity; Tsc1, tuberous sclerosis complex subunit 1; TSC1^DC-KO^, specific ablation of *Tsc1* in the DC compartment; UI, uninfected; WT, wild-type.

To decipher how TSC1 deficiency in DCs leads to T-cell impairment primarily on the CD8^+^ T-cell compartment, we conducted genome-wide RNA sequencing (RNA-seq) analysis on splenic DCs purified from wild-type and TSC1^DC-KO^ mice. It is notable that several gene sets involved in metabolism, antigen presentation, and T-cell regulation were differentially expressed in TSC1^−/−^ DCs (**Fig 4A** and **S5A** and **S5B Fig**). Corroborating the reported role for TSC1-mTORC1 in glycolysis and fatty acid synthesis [28], real-time PCR analyses validated the elevated expression of glycolytic genes solute carrier family 2 member 1 (*Slc2a1* [Glucose transporter 1 (Glut1)]), lactate dehydrogenase A (*Ldha*), and hexokinase 2 (*Hk2*), as well as genes involved in fatty acid synthesis such as sterol regulatory element binding transcription factor chaperone (*Scap*), 3-hydroxy-3-methylglutaryl-coenzyme A reductase (*Hmgcr*), and acetyl-CoA carboxylase alpha (*Acaca*) (encoding ACC1) in TSC1^−/−^ splenic DCs and bone marrow–derived DCs (BMDCs) (**Fig 4B**). Consistent with increased Glut1 expression, BMDCs from TSC1^DC-KO^ mice demonstrated efficient uptake of 2-deoxy-2-[(7-nitro-2, 1, 3-benzoxadiazol-4-yl) amino]-D-glucose (2-NBDG), a fluorescent glucose analogue (**Fig 4C**). In accordance with the elevation of ACC1, pronounced boron-dipyrromethene (BODIPY) staining, and thus lipid accumulation, was associated with TSC1^−/−^ DCs (**Fig 4D**).

Subsequently, we conducted Seahorse assays to examine the metabolic processes in TSC1^−/−^ DCs. By measuring extracellular acidification rate (ECAR), which is indicative of glycolytic rate, we found that the conversion of glucose to lactate was up-regulated in TSC1^−/−^ DCs (**Fig 4E–4G**). Moreover, despite their unaltered mitochondria mass (**S5C Fig**), TSC1^−/−^ DCs displayed marked increase in oxygen consumption rate (OCR), indicative of accelerated OXPHOS and tricarboxylic acid (TCA) cycle (**Fig 4E–4G**). Furthermore, in addition to marked elevation in both basal and maximal OCRs, TSC1^−/−^ DCs also acquired prominent spare respiratory capacity (SRC) (**Fig 4E** and **4F**), which reflects the difference between maximal and basal OCRs. Importantly, etomoxir, but not 2-deoxy-D-glucose (2-DG) or bis-2-(5-phenylacetamido-1, 3, 4-thiadiazol-2-yl) ethyl sulfide (BPTES), inhibited the spare OCR (**S5D Fig**), thus suggesting that TSC1^−/−^ DCs are more prone to use FAO generating energy.

Unexpectedly, TSC1^−/−^ DCs also exhibited impaired expression of cytokines *Il7*, *Il15*, and *Il15Rα*, which have been widely linked to T-cell homeostasis, and antigen-presenting genes histocompatibility 2, K1, K region (*H2-k1*) and histocompatibility 2, D region locus 1 (*H2-d1*), which encode MHC class I molecules histocompatibility 2, K1, K region (H2-K^b^) and histocompatibility 2, D region locus 1 (H2-D^b^), respectively (**Fig 4A** and **4B**). Flow cytometry analyses confirmed that the cell surface presence of MHC-I H2-K^b^ and H2-D^b^, but not of MHC-II, was down-regulated in TSC1^−/−^ DCs (**Fig 4H**). Moreover, both CD8α^+^ and CD11b^+^ DC subsets from the TSC1^DC-KO^ spleen showed reduction in MHC-I but not MHC-II expression (**S5E Fig**). Upon infection with L.M.-OVA, splenic DCs from the wild-type mice showed notable surface presentation of H2-K^b^/OVA_257-264_ (H2-K^b^-SIINFEKL) complexes, indicative of efficient cross presentation of antigens by DCs in vivo (**Fig 4I**). Conversely, flow cytometry analysis barely detected H2-K^b^/OVA_257-264_ complexes on the surface of splenic DCs from TSC1^DC-KO^ mice (**Fig 4I**), demonstrating severely compromised capacity in MHC-I-restricted cross presentation. Hence, transcriptome analyses revealed crucial roles for TSC1 in the control of antigen presentation, cytokine production, and fatty acid synthesis in DCs.

### Impaired expression of H2-K^b^ and IL-7 underlie TSC1^−/−^ DC dysfunction

**Fig 5:**
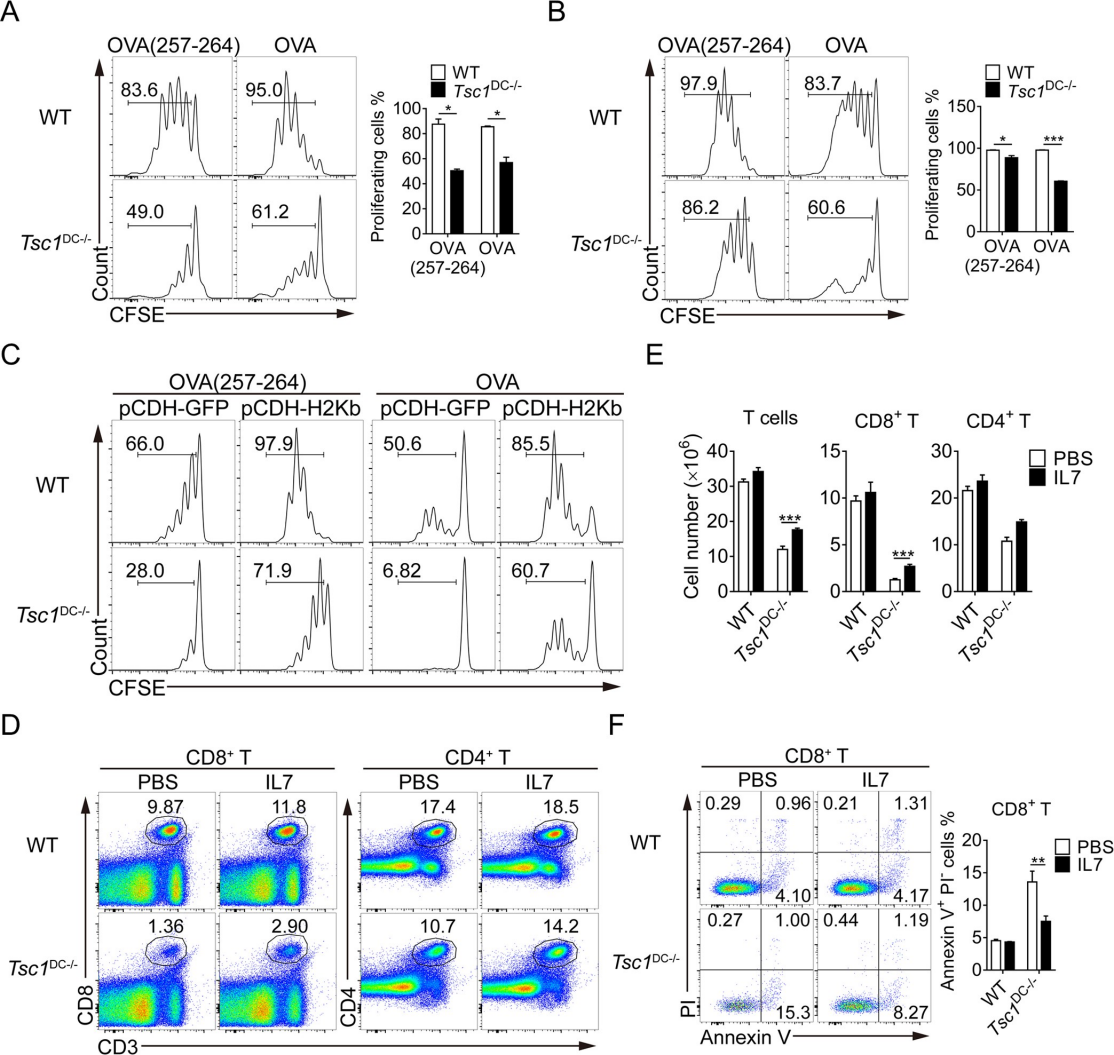
Exogenous MHC-I and IL-7 can rescue CD8^+^ T-cell defects in TSC1^DC-KO^ mice. (A and B) WT and TSC1^DC-KO^ BMDCs (A) or spleen DCs (B) pulsed with 0.1 ng/ml OVA_257-264_ or 0.25 mg/ml OVA for 1 hour and 6 hours, respectively, were cocultured with CFSE-labeled purified OT-I CD8^+^ T cells for 3 days. The proliferation of OT-I CD8^+^ T cells was analyzed by division of CFSE by flow cytometry. This experiment was repeated three times with similar results. The data are presented as means ± SEM (*p < 0.05, ***p < 0.001; analyzed by Student’s *t* test). (C) Control (pCDH-GFP) and H2-K^b^ expressing (pCDH-H2-K^b^) lentiviruses vectors were transfected into BMDCs, and GFP^+^ DCs were sorted. Transfected BMDCs were then pulsed with 0.1 ng/ml OVA_257-264_ or 0.25 mg/ml OVA for 1 hour and 6 hours, respectively, and cocultured with CFSE-labeled OT-I CD8^+^ T cells for 3 days. Cell proliferation indicated by division of CFSE was assessed by flow cytometry. This experiment was repeated once with similar results. (D**–**F) The 6–8-week-old WT and TSC1^DC-KO^ littermates (*n* = 6) were i.p. injected with PBS or IL-7 (10 μg/kg) for 5 days. The percentages (D), cell numbers (E), and apoptotic ratios (F) were analyzed by flow cytometry, respectively. These experiments were repeated twice with similar results. The data are presented as means ± SEM (**p < 0.01, ***p < 0.001; analyzed by Student’s *t* test). Underlying data are available in S1 Data. BMDC, bone marrow–derived DC; CD, cluster of differentiation; CFSE, carboxyfluorescein diacetate succinimidyl ester; DC, dendritic cell; GFP, green fluorescent protein; H2-Kb, histocompatibility 2, K1, K region; i.p., intraperitoneally; IL, interleukin; MHC, major histocompatibility complex; OT, ovalbumin-specific TCR transgenic mouse; OVA, ovalbumin; PBS, phosphate-buffered saline; PI, propidium iodide; Tsc1, tuberous sclerosis complex subunit 1; TSC1^DC-KO^, specific ablation of *Tsc1* in the DC compartment; WT, wild-type.

Considering MHC-I-restricted antigen presentation is essential for CD8^+^ T-cell response, we sought to directly assess antigen presentation in DCs via well-established in vitro assays. Untreated or lipopolysaccharide (LPS)-treated BMDCs were loaded with OVA_257-264_ peptides, and the surface presentation of H2-K^b^/OVA_257-264_ complexes were assessed by flow cytometry. Compared with the wild-type DCs, TSC1^−/−^ DCs showed considerable reduction in surface presentation of H2-K^b^/OVA_257-264_ complexes before and after LPS stimulation (**S6A Fig**). Nevertheless, the ability to uptake dextran was well preserved in TSC1^−/−^ DCs (**S6B Fig**). Hence, TSC1^−/−^ DCs are compromised specifically in antigen presentation, rather than in general processes like endocytosis or phagocytosis. As DC presentation of H2-K^b^/OVA_257-264_ or MHC-II/OVA_323-339_ can lead to ovalbumin-specific TCR transgenic mouse (OT)-I CD8^+^ or OT-II CD4^+^ T-cell proliferation in vitro, we further interrogated antigen presentation in TSC1^−/−^ DCs via the functional assay. As expected, wild-type BMDCs loaded with OVA or OVA_257-264_ peptides were able to drive successive division of carboxyfluorescein diacetate succinimidyl ester (CFSE)-labeled naïve OT-I CD8^+^ T cells (**Fig 5A**). Conversely, TSC1^−/−^ BMDCs loaded with respective antigens were much less efficient in driving OT-I CD8^+^ T cells’ division (**Fig 5A**). It is noteworthy that TSC1^−/−^ BMDCs loaded with OVA_323-339_ were able to trigger OT-II CD4^+^ T-cell proliferation as well as wild-type BMDCs, indicating the intact MHC-II-restricted antigen presentation (**S6C Fig**). Furthermore, splenic TSC1^−/−^ DCs loaded with OVA were also defective in driving OT-I CD8^+^ T-cell proliferation (**Fig 5B**).

To correlate H2-K^b^ reduction with defective cross presentation, we restored H2-K^b^ expression in TSC1^−/−^ DCs via lentiviral transduction (**S6D Fig**) and followed this with functional analysis of antigen presentation. Whereas the exogenous expression of H2-K^b^ only marginally elevated the antigen-presenting ability in wild-type BMDCs, it resulted in prominent augmentation of antigen presentation in TSC1^−/−^ BMDCs (**Fig 5C**). In particular, once loaded with OVA proteins, H2-K^b^-transducted TSC1^−/−^ BMDCs demonstrated a nearly 10-fold increase in antigen cross presentation, reflected by OT-I CD8^+^ T-cell proliferation (**Fig 5C**). Remarkably, H2-K^b^-restored TSC1^−/−^ DCs closely resembled wild-type DCs in terms of triggering CD8^+^ T-cell proliferation, thus underscoring the functional relevance of TSC1-regulated MHC-I expression in DCs.

Similarly, we addressed the functional relevance of IL-7 reduction in TSC1^DC-KO^ mice through intraperitoneal injection of exogenous IL-7 to restore T-cell homeostasis. Interestingly, exogenous IL-7 did not have any significant impact on T cells in the wild-type mice: neither CD8^+^ nor CD4^+^ T-cell numbers were altered following IL-7 injection. In contrast, supplementation with IL-7 exerted a notable effect on T lymphocytes in the TSC1^DC-KO^ mice, whose CD8^+^ T cells were doubled, but was still lower than that in wild-type mice (**Fig 5D** and **5E**). Mechanistically, IL-7 supplementation decreased the percentage of apoptotic CD8^+^ T cells in the TSC1^−/−^ DCs by 2-fold (**Fig 5F**), implicating an important role for DC-derived IL-7 in maintaining homeostatic CD8^+^ T-cell survival.

### Disrupted metabolic–epigenetic program responsible for MHC-I and IL-7 expression

**Fig 6:**
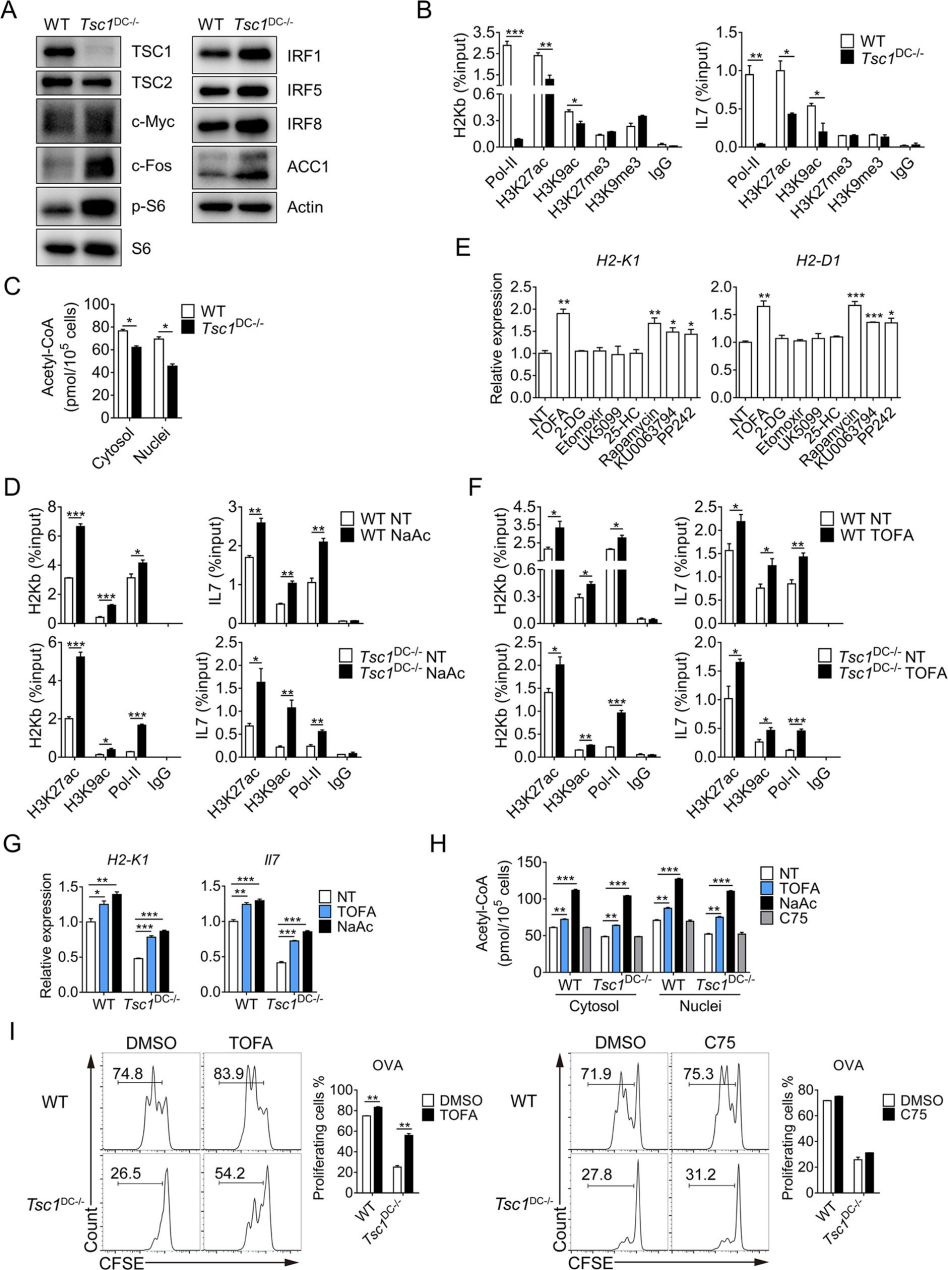
Aberrant fatty acid synthesis impairs histone acetylation and gene expression in DCs. (A) Whole-cell lysates were prepared from WT and TSC1^DC-KO^ splenic DCs, and immunoblotting was performed with indicated antibodies. This experiment was repeated twice with similar results, and the representative data were shown. (B) WT and TSC1^DC-KO^ BMDCs were cross-linked and lysed, followed by immunoprecipitation with anti-p-pol-II (S5), anti-H3K27ac, anti-H3K9ac, anti-H3K27me3, and anti-H3K9me3. Immunoprecipitated *H2k1* and *Il7* promoter DNA fragments were measured by real-time PCR and quantified over respective inputs. This experiment was conducted three times with similar results. The data are presented as means ± SEM (*p < 0.05, **p < 0.01, ***p < 0.001; analyzed by Student’s *t* test). (C) The acetyl-CoA levels in the cytosol and nuclei of WT and TSC1^−/−^ BMDCs were measured by an acetyl-CoA assay kit according to the manufacturer’s instructions. This experiment was conducted twice with similar results. The data are shown as means ± SEM (*p < 0.05, analyzed by Student’s *t* test). (D) WT and TSC1^−/−^ BMDCs were either untreated or treated with 10 mM NaAc for 24 hours and cross-linked, and the cell lysates were immunoprecipitated with indicated antibodies. Immunoprecipitated *H2k1* and *Il7* promoter DNA fragments were measured by real-time PCR and quantified over respective inputs. This experiment was conducted twice with similar results. The data are presented as means ± SEM (*p < 0.05, **p < 0.01, ***p < 0.001; analyzed by Student’s *t* test). (E) TSC1^−/−^ BMDCs were either untreated or treated with indicated inhibitors for 24 hours, and *H2k1* and *H2d1* levels were measured by real-time PCR. This experiment was conducted twice with similar results. The data are presented as means ± SEM (*p < 0.05, **p < 0.01, ***p < 0.001; analyzed by Student’s *t* test). (F) WT and TSC1^−/−^ BMDCs were either untreated or treated with 20 μM TOFA for 24 hours and cross-linked. The cell lysates were immunoprecipitated with indicated antibodies. Immunoprecipitated *H2k1* and *Il7* promoter DNA fragments were measured by real-time PCR and quantified over respective inputs. This experiment was conducted twice with similar results. The data are presented as means ± SEM (*p < 0.05, **p < 0.01, ***p < 0.001; analyzed by Student’s *t* test). (G) WT and TSC1^−/−^ BMDCs were either untreated or treated with 20 μM TOFA or 10 mM NaAc for 24 hours, and total mRNAs were extracted for real-time PCR analysis of *H2k1* and *Il7* expression levels. This experiment was performed twice with similar results. The data are presented as means ± SEM (*p < 0.05, **p < 0.01, ***p < 0.001, analyzed by Student’s *t* test). (H) BMDCs were treated with 20 μM TOFA or 10 mM NaAc or 5 μM C75 for 24 hours, and the amounts of acetyl-CoA in the cytosol and nuclei were measured. This experiment was repeated once with similar results. The data are presented as means ± SEM (**p < 0.01, ***p < 0.001, analyzed by Student’s *t* test). (I) WT and TSC1^−/−^ BMDCs were either untreated or treated with 20 μM TOFA or 5 μM C75 for 24 hours and then pulsed with 0.25 mg/ml OVA for 6 hours. After removal of culture media, the treated BMDCs were washed twice and then cocultured with CFSE-labeled OT-I CD8^+^ T cells for 2 days, and the proliferation of OT-I CD8^+^ T cells was analyzed by flow cytometry. This experiment was conducted twice with similar results. The data are presented as means ± SEM (**p < 0.01, analyzed by Student’s *t* test). Underlying data are available in S1 Data and S1 Raw Images. 2-DG, 2-deoxy-D-glucose; 25-HC, 25-hydroxycholesterol; ACC1, acetyl-CoA carboxylase 1; acetyl-CoA, acetyl-coenzyme A; BMDC, bone marrow–derived DC; c-Fos, FBJ osteosarcoma oncogene; CFSE, carboxyfluorescein diacetate succinimidyl ester; c-Myc, cellular myelocytomatosis; DC, dendritic cell; H2-D1, histocompatibility 2, D region locus 1; H2-K1, histocompatibility 2, K1, K region; H2-K^b^, histocompatibility 2, K1, K region; H3K27ac, acetyl-histone H3 (Lys27); H3K27me3, tri-methyl-histone H3 (Lys27); H3K9ac, acetyl-histone H3 (Lys9); H3K9me3, tri-methyl-histone H3 (Lys9); IgG, immunoglobulin G; Il, interleukin; IRF, interferon regulatory factor; NaAc, sodium acetate; NT, untreated; OT, ovalbumin-specific TCR transgenic mouse; OVA, ovalbumin; S6, ribosomal protein S6; Pol-II, RNA polymerase II; TOFA, 5-(tetradecyloxy)-2-furoic acid; Tsc1, tuberous sclerosis complex subunit 1; Tsc2, tuberous sclerosis complex subunit 2; TSC1^DC-KO^, specific ablation of *Tsc1* in the DC compartment; WT, wild-type.

To understand how TSC1 deficiency leads to the reduction in IL-7 and H2-K^b^ expression, we examined transcriptional factors and epigenetic markers relevant to both genes [32]. We first examined the expression of a variety of transcriptional factors but found that certain transcriptional activators such as FBJ osteosarcoma oncogene (c-fos), IFN regulatory factors (IRFs), and c-Myc were actually elevated in TSC1^−/−^ DCs, whereas others such as nuclear factor kappa B (NFκB) and cAMP responsive element binding proteins (CREBs) were unaltered (**Fig 6A** and **S7A Fig**). These results may be helpful to explain why genes involved in fatty acid synthesis were up-regulated, but they could not directly address how the defective gene expression occurred. We therefore conducted chromatin immunoprecipitation (ChIP)-quantitative PCR (qPCR) to examine the epigenetic markers associated with *Il7* and *H2-k1*. The presence of a variety of histone methylation markers, especially tri-methyl-histone H3 (Lys27) (H3K27me3) and tri-methyl-histone H3 (Lys9) (H3K9me3), in the promoter regions of *Il7* and *H2-k1* genes were largely comparable between wild-type and TSC1^−/−^ DCs (**Fig 6B**). However, the enrichment of acetylation markers acetyl-histone H3 (Lys27) (H3K27ac) and acetyl-histone H3 (Lys9) (H3K9ac) within *Il7* and *H2-kb* promoters was substantially reduced in the TSC1^−/−^ DCs (**Fig 6B**). Given the established correlation between acetyl-CoA abundance and histone acetylation, we sought to quantitatively measure cellular acetyl-CoA levels. Remarkably, decreased acetyl-CoA was detected in both the cytosolic and nuclear compartments of TSC1^−/−^ DCs (**Fig 6C**). To determine whether the reduction in nuclear acetyl-CoA was accountable for the decrease in histone acetylation, we supplemented both wild-type and TSC1^−/−^ DCs with abundant acetate, which could convert into acetyl-CoA by acyl-CoA synthetase short chain family member 2 (ACSS2) in the cytosolic/nuclear compartments [25]. Notably, replenishing acetyl-CoA with acetate elevated the enrichment of epigenetic markers H3K27ac and H3K9ac in *Il7* and *H2-k1* promoters, especially in the TSC1^−/−^ DCs (**Fig 6D**). These results revealed an intimate association between acetyl-CoA production and histone acetylation in the regulation of a DC-specific gene program.

Given the widespread association between metabolic reprograming and acetyl-CoA fluctuations in the regulation of gene expression [22,23], we subsequently examined which metabolic processes might be responsible for the altered epigenetic regulation on DC-specific genes. To this end, we screened a panel of inhibitors targeting various metabolic checkpoints by assessing their effects on the expression of MHC-I molecules H2-K^b^ and H2-D^b^. Corroborating the critical role of TSC1-mTORC1 in DC function (**Fig 2A**), blockade of mTOR with rapamycin, KU0063794, and PP242 increased H2-K^b^ and H2-D^b^ expression in BMDCs (**Fig 6E**) but decreased MHC-II expression (**S7B Fig**). However, 2-DG, which targets hexose kinase HK, did not alter either H2-K^b^ or H2-D^b^ expression (**Fig 6E** and **S7C Fig**), indicating a dispensable role for glycolysis in this process. Moreover, dampening FAO by etomoxir also failed to exert any significant impact on MHC-I expression. Remarkably, blockade of ACC1 by 5-(tetradecyloxy)-2-furoic acid (TOFA) elevated H2-K^b^ and H2-D^b^ expression (**Fig 6E**) but had no impact on MHC-II molecules (**S7B Fig**). In contrast, blockade of other enzymes required for fatty acid synthesis, such as Acly and fatty acid synthase (Fasn) with Acly inhibitor (Aclyi) and C75, respectively, did not have the same effect as TOFA (**S7D** and **S7E Fig**). As all these inhibitors similarly target fatty acid synthesis, their differential effects on MHC-I argue against a crucial role for fatty acid synthesis per se in the regulation of MHC-I expression.

Notably, ACC1 expression was elevated in TSC1^−/−^ DCs, as demonstrated by real-time PCR and immunoblotting (**Fig 4A** and **4B** and **Fig 6A**). It is plausible that highly active ACC1 led to consumption of acetyl-CoA for malonyl-coenzyme A (malonyl-CoA) and subsequent fatty acid synthesis. Conceivably, blockade of ACC1 could result in acetyl-CoA accumulation and thus possible increase in histone acetylation. Indeed, TOFA treatment increased the abundance of H3K27ac and H3K9ac in *Il7* and *H2-k1* promoters and concomitant recruitment of polymerase II (**Fig 6F**). Consequently, the expression of IL-7 and H2-Kb were up-regulated by TOFA or acetate (**Fig 6G**). Notably, treatment with either TOFA or acetate but not C75 led to increased acetyl-CoA in the cytosolic and nuclear compartments of wild-type and TSC1^−/−^ DCs (**Fig 6H**). Correspondingly, TSC1^−/−^ DCs pretreated with TOFA showed increased capacity in antigen presentation of both OVA proteins and OVA_253-264_ peptides (**Fig 6I** and **S7E Fig**). In contrast, TSC1^−/−^ DCs pretreated with C75 had slightly elevated cross presentation of OVA (**Fig 6I**) but similar antigen presentation of OVA_253-264_ peptides (**S7E Fig**). These results demonstrate that blockade of ACC1 is the most efficient way to rescue the metabolic dysfunction in TSC1^−/−^ DCs.

**Fig 7:**
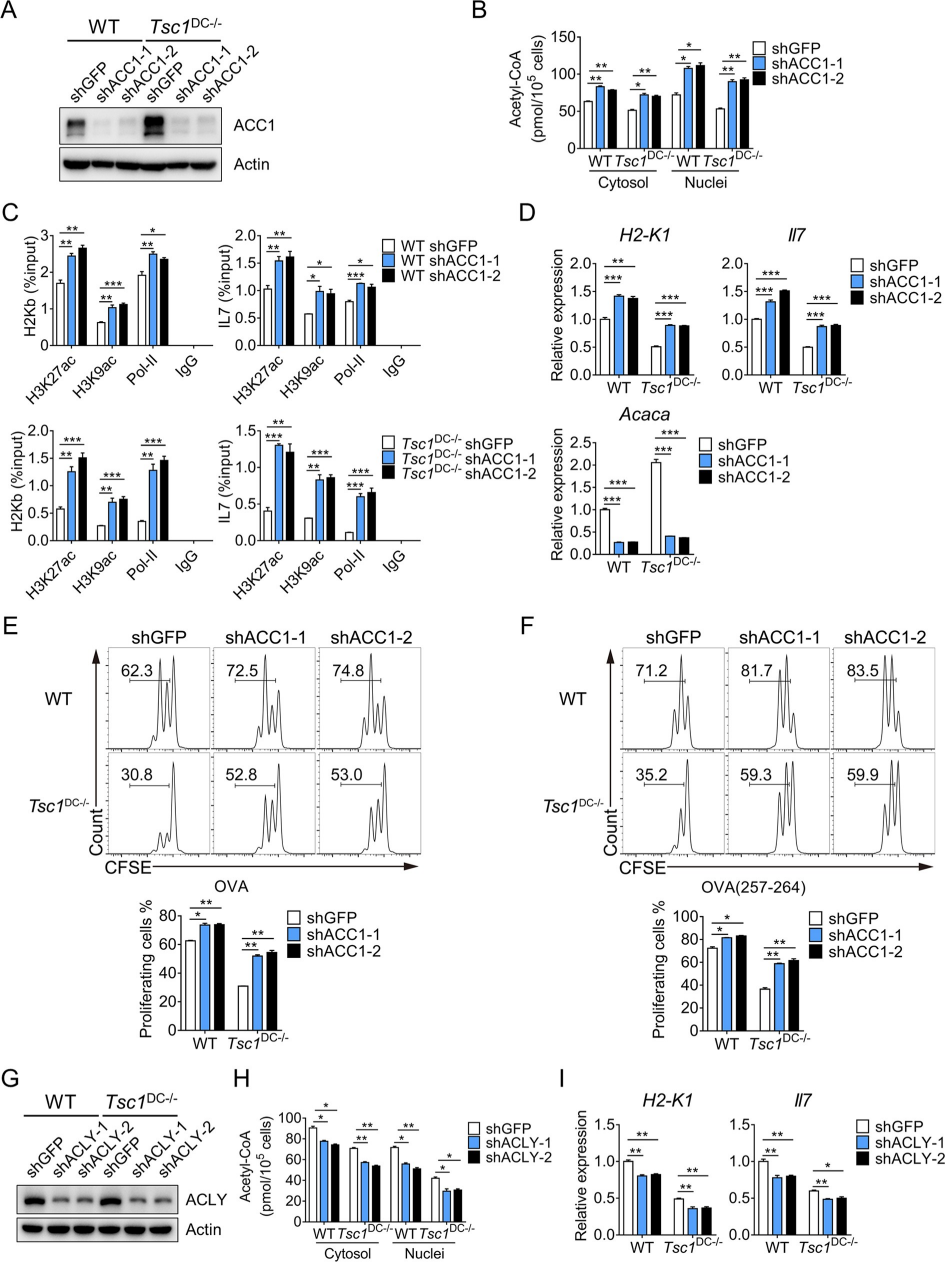
ACC1 controls acetyl-CoA production and MHC-I/IL-7 expression in DC. (A) Whole-cell lysates prepared from control (shGFP) and ACC1-knockdown BMDCs were immunoblotted with indicated antibodies. (B) The acetyl-CoA levels in the cytosol and nucleus of control (shGFP) and ACC1-knockdown BMDCs were measured. This experiment was repeated once with similar results. The data are presented as means ± SEM (*p < 0.05, **p < 0.01, analyzed by Student’s *t* test). (C) Control and ACC1-knockdown BMDCs were cross-linked, and the cell lysates were immunoprecipitated with indicated antibodies. Immunoprecipitated *H2k1* and *Il7* promoter DNA fragments were measured by real-time PCR and quantified over respective inputs. This experiment was conducted twice with similar results. The data are presented as means ± SEM (*p < 0.05, **p < 0.01, ***p < 0.001; analyzed by Student’s *t* test). (D) The expression of *H2k1*, *Il7*, and *Acaca* were measured by real-time PCR. This experiment was performed twice with similar results, and the data are presented as means ± SEM (**p < 0.01, ***p < 0.001, analyzed by Student’s *t* test). (E and F) Control and ACC1-knockdown BMDCs were pulsed with 0.25 mg/ml OVA (E) or 0.1 ng/ml OVA_257-264_ (F) for 6 hours and 1 hour, respectively. The treated BMDCs were cocultured with CFSE-labeled OT-I CD8^+^ T cells for 2 days, and the proliferation of OT-I CD8^+^ T cells was analyzed by flow cytometry. This experiment was conducted twice with similar results. The data are presented as means ± SEM (*p < 0.05, **p < 0.01, analyzed by Student’s *t* test). (G) Whole-cell lysates prepared from control and *Acly*-knockdown BMDCs were probed with indicated antibodies. (H) The acetyl-CoA levels in the cytosol and nuclei of control and *Acly*-knockdown BMDCs were measured. This experiment was repeated once with similar results. The data are presented as means ± SEM (*p < 0.05, **p < 0.01, analyzed by Student’s *t* test). (I) Total mRNAs of control and *Acly*-knockdown BMDCs were extracted for real-time PCR analysis of *H2k1* and *Il7* expression levels. This experiment was performed twice with similar results. The data are presented as means ± SEM (*p < 0.05, **p < 0.01, analyzed by Student’s *t* test). Underlying data are available in S1 Data and S1 Raw Images. Acaca, acetyl-CoA carboxylase alpha; ACC1, acetyl-CoA carboxylase 1; acetyl-CoA, acetyl-coenzyme A; Acly, ATP-citrate lyase; BMDC, bone marrow–derived DC; CD, cluster of differentiation; CFSE, carboxyfluorescein diacetate succinimidyl ester; GFP, green fluorescent protein; H2-K1, histocompatibility 2, K1, K region; H2-K^b^, histocompatibility 2, K1, K region; H3K27ac, acetyl-histone H3 (Lys27); H3K9ac, acetyl-histone H3 (Lys9); IgG, immunoglobulin G; IL, interleukin; MHC, major histocompatibility complex; OVA, ovalbumin; Pol-II, RNA polymerase II; sh, short hairpin; Tsc1, tuberous sclerosis complex subunit 1; WT, wild-type.

**Fig 8:**
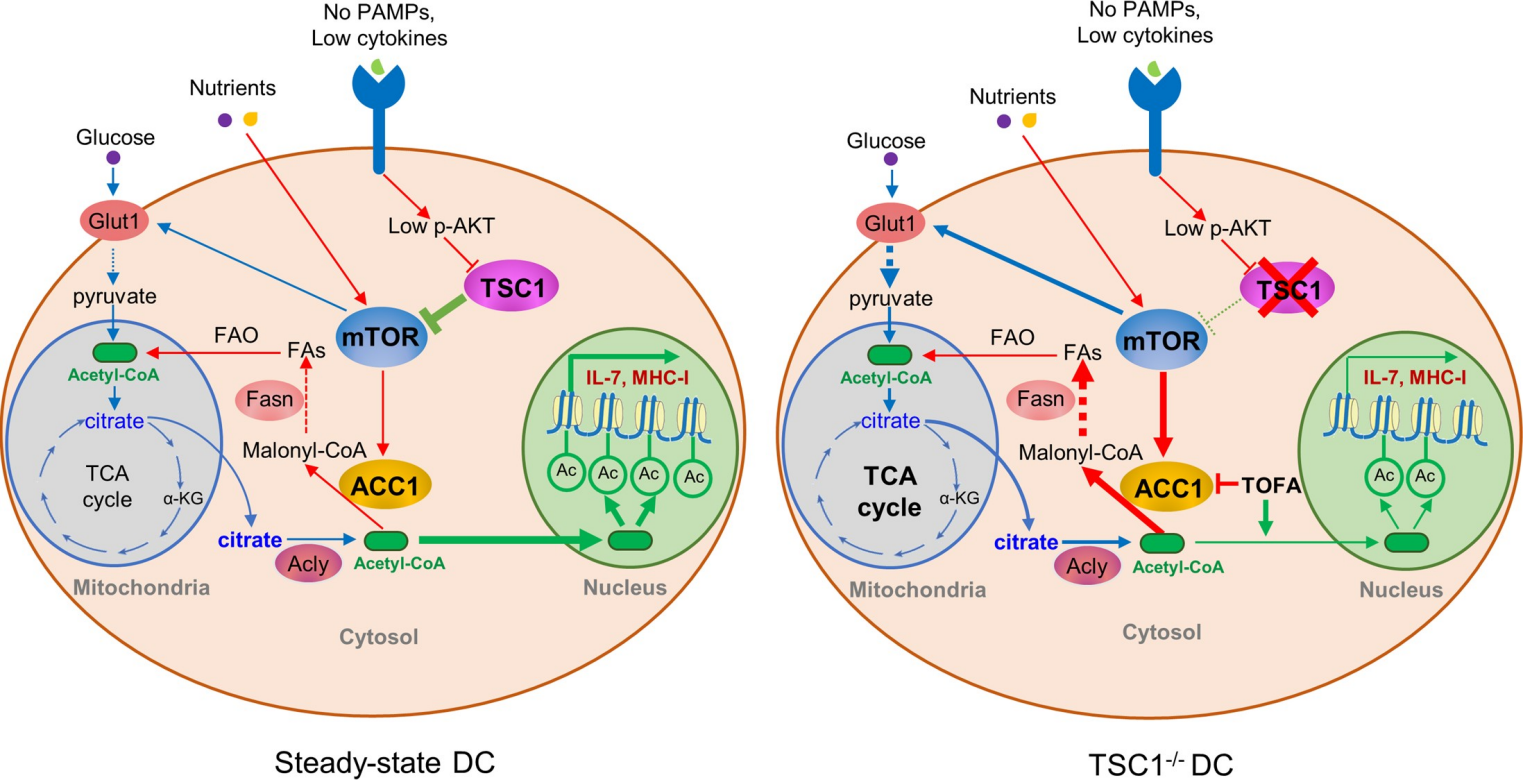
A work model illustrating the reciprocal regulation of metabolism and epigenetics in the steady-state DCs. Because of the lack of PAMPs and inflammation, steady-state DCs receive only tonic signals from the environment and thus tune down mTOR activation and fatty acid synthesis through TSC1/TSC2, ensuring adequate MHC-I and IL-7 expression. In the absence of TSC1, mTOR becomes hyperactivated, thus propelling exuberant ACC1-driven fatty acid synthesis. Consequently, only scarce acetyl-CoAs are available for histone acetylation and selective gene expression in the steady-state TSC1^−/−^ DCs. α-KG, α-ketoglutarate; ACC1, acetyl-CoA carboxylase 1; acetyl-CoA, acetyl-coenzyme A; Acly, ATP-citrate lyase; DC, dendritic cell; FA, fatty acid; FAO, FA oxidation; Fasn, FA synthase; Glut1, glucose transporter 1; IL, interleukin; malonyl-CoA, malonyl-coenzyme A; MHC, major histocompatibility complex; mTOR, mechanistic target of rapamycin; p-AKT, phosphorylated AKT; PAMP, pathogen-associated molecule pattern; TCA, tricarboxylic acid, TSC1, tuberous sclerosis complex subunit 1; TSC2, tuberous sclerosis complex subunit 2.

Next, we took a genetic approach to validate the role of ACC1 in the regulation of acetyl-CoA and *H2k1*/*Il7* expression in DCs. Upon knocking down the expression of ACC1 (**Fig 7A**), we observed increased acetyl-CoA levels in the cytosolic and nuclear compartments of both wild-type and TSC1^−/−^ DCs (**Fig 7B**). Consistently, ACC1 knockdown led to elevated histone acetylation markers H3K27ac and H3K9ac on the promoters of *H2k1* and *Il7* (**Fig 7C**). Consequently, DC-mediated OT-I CD8^+^ T-cell proliferation was considerably augmented in ACC1-knockdown DCs, correlating with increased H2-Kb and IL7 expression (**Fig 7D–7F**). On the other hand, short hairpin RNA (shRNA) knockdown of *Alcy* led to reduced acetyl-CoA production and impaired expression of H2-kb and IL-7 in DCs (**Fig 7G–7I**). Together, these results identify ACC1 as a key checkpoint where metabolic and epigenetic pathways intersect (**Fig 8**).

## Discussion

Recent findings have placed the multifaceted TSC1-mTORC1 pathway at the center of DC biology. Nevertheless, the full spectrum of TSC1-mTOR function in diverse DC subsets has yet to be unveiled, and the complex mechanisms involved remain very much underappreciated. In this study, we uncovered a central role for TSC1-mTOR in programing DC metabolism, a process integral to homeostatic DC–T cell interaction. Whereas DCs have been conventionally linked to T-cell priming and activation, its role in immune tolerance and homeostasis has also emerged. Upon entering the periphery, naïve CD8^+^ T cells rely on two major signals for long-term survival—i.e., the tonic TCR signaling triggered by self-peptide/MHC-I complexes and the intermittent IL-7 signaling [9,13]. Although multiple cell types are capable of supplying both signals [10–12,15,33–36], our data strongly support the assertion that DCs are the key cellular source providing survival signals for CD8^+^ T cells. Metabolic programing has pivotal roles in immune cell function, yet how metabolism impinges on epigenetics to influence gene expression has been less explored. In this regard, the metabolic–epigenetic coupling mechanism revealed by this study may serve as a paradigm for further understanding of metabolic regulation in the immune system.

Because of its unique ability to coordinate gene transcription/translation with cell metabolism, it is not surprising that the TSC1-mTOR pathway has been implicated in DC development and function. Whereas ablation of TSC1 through inducible Cre-estrogen receptor (CreER) led to marked reduction in all the DC subsets, irrespective of their anatomic locations [28], DC-specific ablation of late endosomal/lysosomal adaptor, MAPK and MTOR activator (LamTOR), mTOR, or Raptor via CD11c-Cre caused selective loss of CD103^+^ DCs and Langerhans cells only in certain nonlymphoid tissues including lung and skin [19,37–39]. In this study, DC-specific ablation of TSC1 through Cd11c-Cre did not cause widespread defects in DC development either; the resident DC subsets in the secondary lymphoid organs were well reserved. However, we did observe relatively subtle reduction of the migratory DCs within the pLNs of TSC1^DC-KO^ mice. Whether the reduction in migratory DCs might have contributed to the decrease in CD8^+^ T cells remains an open question; however, the fact that the CD4^+^ T cells were normally present in the LNs argue against this scenario. It has been reported that the tonic inhibitor of kappaB kinase (IKK)β activity is involved in DC migration to the draining LNs [40]; however, TSC1^−/−^ DCs demonstrated increased IKKβ activation upon LPS stimulation instead [29]. As TSC1^−/−^ DCs also display normal surface CCR7, the mechanism underlying their migration defect has yet to be determined.

Although TSC1 expression may not be absolutely required for DC development and survival, it can be involved in various DC functions. Previous studies have shown that TSC1-deficient DCs are defective in CD4^+^ T-cell priming and differentiation, in part because of compromised MHC-II and IL-12 expression [28,29]. Interestingly, we did not see any major defect in the CD4^+^ T-cell compartment that is contingent on TSC1 deficiency in DCs. Instead, TSC1 deficiency in DCs profoundly affected the CD8^+^ T-cell compartment, leading to substantial reduction in both naïve and memory–phenotype CD8^+^ T cells in the steady state, and severely compromised CD8^+^ T-cell activation in response to L.M. infection and melanoma challenge. Mechanistically, our data suggest a scenario that defective expression of MHC-I and cytokines such as IL-7 in the DC compartment might have contributed to the overt apoptosis in CD8^+^ T cells. Although the current consensus is that naïve CD8^+^ T cells need continuous but intermittent engagement with IL-7 and MHC-I in order to maintain long-term survival in the periphery, it is the cellular source for both signals that has been under debate. Because MHC-I molecules are ubiquitously expressed, both immune cells and nonimmune cells have been shown to be able to present self-peptide and promote naïve CD8^+^ T-cell survival and/or proliferation [10,11,41]. However, there are also circumstances in which DC presentation of self-antigen can be mandatory for CD8^+^ T-cell survival [35,42]. Indeed, DCs, particularly XCR1^+^ DCs in the secondary lymphoid organs and CD103^+^ DCs in the peripheral tissues, possess a unique ability to cross-present antigens captured from damaged or apoptotic cells through micropinocytosis or efferocytosis [34,43,44]. With abundant enzymes specialized in precision digestion and processing antigens, DCs are also better equipped for generating self-peptides fitting into MHC-I molecules. In this regard, it is plausible that DCs may be able to present a much broader range of antigens than non-DCs. Indeed, DCs, especially migratory DCs, are known to constantly survey peripheral tissues, engulfing apoptotic cells, processing self-antigens, and cross-presenting them to naïve CD8^+^ T cells in the LNs. Therefore, DCs may have a nonredundant role in cross-presenting tissue antigens to naïve CD8^+^ T cells with special TCR repertoires, thus supporting the abundance and breadth of naïve CD8^+^ T-cell population.

IL-7 is a pleiotropic cytokine required not only for CD8^+^ but also for CD4^+^ T-cell survival. However, it remains a great challenge to unequivocally determine the cellular sources for IL-7, which is generally present at low abundance. Whereas a broad range of epithelia and fibroblasts can produce ample IL-7, DCs also express a decent amount of IL-7 [14,15,45]. Importantly, DC production of IL-7 can be crucial for T-cell survival and proliferation [12]. As T-cell numbers are generally kept steady, exogenous administration of IL-7 did not have a significant impact on T-cell numbers in the wild-type mice. However, supplementation with IL-7 exerted a notable effect on T cells in the TSC1^DC-KO^ mice, increasing CD8^+^ T cells by 2-fold. These data suggest that DC-produced IL-7 also contributes to the bulk amounts of IL-7. Intriguingly, two studies using CD11c-diphtheria toxin A (DTA) to achieve DC depletion revealed that loss of DCs did not have major effects on T-cell development or homeostasis [46,47], albeit a notable increase in CD4^+^/CD8^+^ ratio was observed in one study [47]. However, our data indicate that the presence of functional defective DCs could impair CD8^+^ T-cell homeostasis. Our finding is consistent with the report that ablation of MHC-II in DCs compromised CD4^+^ T-cell homeostasis in the spleen [48], even though depletion of DCs did not generate such effects [46]. To reconcile these disparate findings, we propose that when DCs are absent, other cells could presumably fill the void and still manage to maintain T-cell homeostasis. Conversely, when DCs are present but functionally corrupted, they would be still poised to engage T cells but incapable of providing adequate help signals. As DCs are the favorite targets for T cells, other cells would have very limited access to T cells under such circumstances. Conceivably, occupying CD8^+^ T cells but failing to provide the intermittent IL-7 and TCR signals may be the key cause of impaired CD8^+^ T-cell response in the TSC1^DC-KO^ mice. In this regard, we propose that CD8^+^ T-cell homeostasis is contingent on the MHC-I/self-peptide and IL-7 signals from the DC compartment.

In addition to host defense, DC has been widely associated with immune homeostasis by sensing environmental cues [2,4]. In line with this notion, TSC1-mTOR has been recognized as one of the core modules responsible for the detection of environmental cues, including nutrients, growth factors, and PAMPs [20,27]. How TSC1-mTOR-controlled cell metabolism works together with gene transcription/translation program that enables DC for immune homeostasis can be complex and context dependent. Whereas TLR-stimulated DCs rapidly elevate glycolysis and fatty acid synthesis for ER and Golgi expansion [18], overaccumulation of lipids can dampen cross presentation as well [49,50]. In the steady-state DCs, glycolysis and OXPHOS are relatively low to accommodate their basic energetic and biosynthetic demands, although CD103^+^ DCs demonstrate higher metabolic activity than CD11b^+^ DCs do [21]. However, in the absence of TSC1, we found that the steady-state DCs carry out active glycolysis and accelerated fatty acid synthesis, thus showing metabolic features fitting into activated DCs rather than steady-state DCs. One of the consequences of fatty acid synthesis is lipid accumulation, which has been implicated in dampening cross presentation of antigens [49,50]; another consequence of fatty acid synthesis is the consumption of cytosolic acetyl-coA, a cofactor highly demanded in the nucleus for histone acetylation and epigenetic regulation of immune gene expression [25,26]. In line with these assumptions, TSC1^−/−^ DCs indeed displayed high amounts of lipids in the cytosol but low amounts of acetyl-CoA in the nucleus. Although Alcy, Acc1, and Fasn are all required for fatty acid synthesis, only blocking ACC1 by TOFA or shRNA rescued H2-K^b^ and IL-7 expression, thus arguing against the notion that lipid accumulation might be responsible for the dysfunction of TSC1^−/−^ DCs. Instead, our data strongly suggest that exhaustion of cytosolic acetyl-CoA by accelerated fatty acid synthesis might have attributed to impaired DC function. In support of this notion, we observed reduced H3K27ac and H3K9ac in the promoters of *H2k1* and *Il7* genes in TSC1^−/−^ DCs, and supplementation with acetate or treatment with TOFA was able to restore the histone acetylation markers. Therefore, this study presents a paradigm highlighting the unique metabolic–epigenetic coupling in immune regulation.

In conclusion, our data support the notion that metabolic programing is integral to DC function and reveal a special metabolic–epigenetic coupled program in imprinting DCs for T-cell homeostasis. We propose that the TSC1-mTOR pathway may play central roles in defining specific DC functions via integration of environmental cues such as nutrients and tissue factors with inflammatory signals like PAMPs and cytokines. We believe that the complex interplay between metabolism and epigenetics has broad implications in autoimmunity and host defense.

## Methods

### Ethics statement

All of the mice were bred and maintained in a specific pathogen–free animal facility at Institut Pasteur of Shanghai. All the procedures were conducted in compliance with a protocol approved by the Institutional Animal Care and Use Committee at Institut Pasteur of Shanghai, licensed by the Science and Technology Commission of Shanghai Municipality (SYXK-2018-0039).

### Mice

*Tsc1* floxed [51], *mTor* floxed [52], and *Rptor* floxed [53] mice; *Cd11c*-cre [54] and *LysM*-cre mice [55]; and OT-I and OT-II TCR transgenic mice [56,57] were obtained from Jackson Laboratories. In all experiments, littermates carrying floxed alleles but without Cre recombinase were used as controls (WT).

### Plasmids and reagents

The H2-K^b^ was amplified from C57BL/6 splenocytes and cloned into a pCDH-GFP vector by enzymes EcoRI and BamHI (NEB). The *Acaca* and *Acly* shRNAs were cloned into a pLKO.1-GFP vector. LPS, OVA_257-264_, OVA_323-339_, and OVA were purchased from InvivoGen. TOFA, C75, 2-DG, 2-NBDG, etomoxir, and BMS-303141 were purchased from Cayman Chemical. Rapamycin, KU0063794, PP242, and BPTES were from Selleck. Mitotracker and BODIPY were purchased from Invitrogen. UK5099, 25-HC, TMR-dextran, oligomycin, fluoro-carbonyl cyanide phenylhydrazone (FCCP), rotenone, and antimycin A were from Sigma. Antibodies for TSC1 (#4906), TSC2 (#4308), c-Myc (#5605), c-Fos (#2250), p-S6 (#4858), S6 (#2317), IRF1 (#8478), IRF5 (#4950), ACC1 (#3662), H3K9ac (#9649), p-ACLY (#4331), ACLY (#4332), p-IκBα (#2859), p-IKKα/β (#2697), p65 (#8242), and CREB (#9197) were from Cell Signaling Technology. Antibodies against H3K27ac (ab4729), H3K27me3 (ab6002), H3K9me3 (ab8898), and IKKα/β (ab178870) were purchased from Abcam. Antibody for IκBα (SC371) was from Santa Cruz Biotechnology. Antibody against GAPDH (AP0063) was from Bioworld.

### Preparation of BMDCs and splenic DCs

Bone marrow cells were isolated from femurs and tibias of 6–10-week-old C57BL/6 mice, and red blood cells were lysed using ACK lysis buffer (0.15 M NH_4_Cl, 1 mM KHCO_3_, and 0.1 mM Na_2_EDTA [pH 7.3]). Bone marrow cells were then seeded at a concentration of 10^6^ cells/ml with RPMI 1640 medium (Invitrogen) containing 10% FBS (HyClone), 20 ng/ml GM-CSF (Peprotech), 10 ng/ml IL-4 (Peprotech), 2 mM L-glutamine (Invitrogen), and 200 μM β-mercaptoethanol (Invitrogen), and the culture medium was replenished every 2 days as described previously [58]. On day 9, the nonadherent cells were collected by centrifugation and then resuspended in fresh medium for use.

For spleen DCs, spleens were minced and digested with 1 mg/ml collagenase IV (Invitrogen) in RPMI 1640 medium containing 10% FBS at 37°C for 30 minutes. Digested cells were then passed through 40-μM cell strainers (BD Biosciences) and incubated with anti-CD11c microbeads according to manufacturer’s instructions (Miltenyi Biotec). The enriched cDCs were then labeled with anti-CD11c and anti-MHC-II antibodies for FACS sorting, and the CD11c^+^MHC-II^+^ population was harvested.

### Lentivirus preparation and infection

The lentiviral vector pCDH-GFP expressing H2-K^b^ or pLKO.1-shACC1 or pLKO.1-shACLY were transiently transfected into HEK293T cells along with packaging plasmids (Δ8.91/VSV-G) at a ratio of pCDH/pLKO.1:Δ8.91:VSVG = 4:3:2, and virus-containing medium was harvested in 48 hours. On day 2 of BMDC differentiation, lentiviruses were added to the BMDCs and incubated for 12 hours. Subsequently, virus-containing medium was removed, and BMDCs were further differentiated for 7 days as described [59]. On day 9, GFP-positive BMDCs were sorted by FACS for further experiments.

### RNA preparation and quantitative real-time PCR

Total RNAs from cells were extracted with TRIzol reagent according to the manufacturer’s instructions (Invitrogen). cDNAs were reverse transcribed from 0.5–1 μg total RNAs with a PrimeScript RT-PCR Kit (Takara). Real-time PCR was conducted with a PrimeScript RT reagent Kit (Takara) on an ABI 7900HT Fast Real-Time PCR machine. The relative expression of genes was quantitatively normalized against the expression of *GAPDH* by the ΔΔCT method. All real-time PCR primers used in this study are shown in **S1 Table**.

### Flow cytometry

Fluorochrome-labeled antibodies for CD8 (53–6.7), CD11b (M1/70), PDCA1 (eBio927), B220 (RA3-6B2), SIRPα (P84), H2-K^b^ (AF6-88.5.5.3), IFNγ (XMG1.2), TNFα (MP6-XT22), H2-K^b^/OVA_257-264_ complexes (25-D1.16), CD80 (16-10A1), CD86 (GL1), CD40 (1C10), CCR7 (4B12), and IL7Rα (A7R34) were from eBioscience. Antibodies for CD3 (145-2C11), CD4 (GK1.5), CD11c (N418), MHC-II (M5/114.15.2), CD103 (M290), CD44 (IM7), and KLRG1 (2F1) were from BD Biosciences. Antibodies for CD62L (MEL-14), XCR1 (ZET), and H2-D^b^ (KH95) were from BioLegend. H2-K^b^/OVA_257-264_ tetramer was purchased from MBL. All antibodies were tested for their specificities with respective isotype controls. For cell surface marker staining, cells were incubated with specific antibodies for 30 minutes on ice, followed by washing with MACS buffer twice. For intracellular cytokine staining, splenocytes were stimulated with OVA_257-264_ for 5 hours in the presence of brefeldin A before staining according to the manufacturer’s instructions (BD Biosciences). Annexin V, PI, and BrdU staining were conducted according to the manufacturer’s instructions (eBioscience and BD Biosciences). All the samples were processed with an LSR-Fortessa flow cytometer (BD), and the data were analyzed by FlowJo software (Tree Star).

### Western blotting

Cells were lysed in lysis buffer (50 mM Tris [pH 7.4], 150 mM NaCl, 1% Triton X-100, and 1 mM EDTA [pH 8.0]) supplemented with protease inhibitor cOmplete mini (Roche) and 1 mM PMSF, 1 mM Na_3_VO_4_, and 1 mM NaF for 30 minutes on ice, and cell debris was removed by centrifugation at 13,000 rpm for 15 minutes. After being boiled for 5 minutes, the samples were separated by 10% SDS-PAGE and were transferred to a PVDF membrane and then probed with the appropriate antibodies.

### Cell fractionation

To obtain cytosolic and nuclear fractions, cells were first resuspended in hypotonic buffer (10 mM HEPES [pH 7.6], 1.5 mM MgCl_2_, 10 mM KCl, and 1 mM EDTA) supplemented with protease inhibitor cOmplete mini (Roche) and 1 mM PMSF, 1 mM Na_3_VO_4_, and 1 mM NaF and gently homogenized by pipetting and lysed on ice for 15 minutes. After centrifugation at 3,000 rpm for 5 minutes, the supernatant was collected as crude cytosolic fraction; the pellet was washed twice in hypotonic buffer with gentle pipetting and further lysed in high-salt buffer (20 mM HEPES [pH 7.6], 500 mM NaCl, 1.5 mM MgCl_2_, and 1 mM EDTA) supplemented with protease inhibitors on ice for 20 minutes to obtain crude nuclear fraction. Crude cytosolic and nuclear fractions were further centrifuged at 13,000 rpm for 15 minutes to remove debris, and the supernatants were collected as cytosolic and nuclear fractions, respectively.

### L.M.-OVA infection

L.M.-OVA was cultured in tryptone soya broth (TSB) medium (OXOID), and the CFU was determined by OD600 absorbance (OD600 = 1 was considered as 10^9^ CFU/ml). The mice were either intravenously (i.v.) injected with 5 × 10^5^ CFU of L.M.-OVA (and survival was monitored daily) or i.v. injected with 10^4^ CFU of L.M.-OVA, and the spleens were isolated after 7 days for intracellular staining of IFNγ/TNFα and tetramer detection of OVA-specific CTL response as described in the following. Infected mice were humanely killed, and spleens were isolated and homogenized into single cells. Splenic cells were then plated onto 96-well plates (1 × 10^6^ cells per well) in RPMI 1640 medium containing 10% FBS. Following restimulation with OVA_257-264_ for 48 hours, supernatants were collected, and the secreted amounts of IFNγ and TNFα were measured by ELISA.

### Tumor model

B16-OVA melanoma cells were cultured in DMEM supplemented with 10% FBS. The wild-type and TSC1^DC-KO^ littermates were injected subcutaneously with 5 × 10^5^ B16-OVA cells in the right flank. Tumor sizes were measured with calipers, and tumor volumes were calculated using the following formula: length × width × depth × 1/2.

### Antigen presentation assays

For in vitro assays, CD8^+^ T cells from OT-I mice or CD4^+^ T cells from OT-II mice were purified by CD8 or CD4 microbeads (Miltenyi Biotec) and labeled with 1 μM CFSE (Invitrogen) for 10 minutes at room temperature. Purified T cells were cocultured with BMDCs or splenic DCs pulsed with OVA_257-264_, OVA_323-339_, or OVA, respectively, at a ratio of DC:T cell = 1:5 in the round-bottomed 96-well plates for 2–3 days. For analysis of OVA_257-264_ loaded on BMDCs, WT and TSC1-deficent BMDCs were untreated or stimulated with 100 ng/ml LPS overnight, followed by incubation with 100 ng/ml OVA_257-264_ for 1 hour. The cell surface H2-K^b^/OVA_257-264_ complexes were stained with monoclonal 25-D1.16 antibody and analyzed by flow cytometry. For in vivo assays, mice were i.v. infected with 10^5^ CFU of L.M.-OVA. After 3 days, spleens were harvested and homogenized into single cells. The presence of H2-K^b^/OVA_257-264_ complexes on splenic DCs were analyzed by flow cytometry accordingly.

### Cell metabolism assays

Cell metabolism was analyzed using an XF-24 Extracellular Flux Analyzer (Seahorse Bioscience). BMDCs or splenic DCs were plated onto XF-24 cell culture plates (the plates were first pretreated with 1 mg/ml poly-D-lysine at 37°C for 1 hour for efficient cell adhesion) (2 × 10^5^ cells/well for BMDCs or 5 × 10^5^ cells/well for splenic DCs) in unbuffered DMEM (Seahorse Bioscience) containing 10% FBS, 2 mM L-glutamine, and 1 mM sodium pyruvate (Sigma). For mitochondrial fitness tests, OCR was measured in response to 1 μM oligomycin, 1.5 μM FCCP, 100 nM rotenone plus 1 μM antimycin A, and 200 μM etomoxir or 100 mM 2-DG or 10 μM BPTES. For glycolysis stress tests, ECAR was measured in response to 10 mM glucose, 1 μM oligomycin, and 100 mM 2-DG.

### RNA-seq and analysis

Total mRNAs were obtained from FACS-sorted splenic DCs, and the integrity was analyzed by a Bioanalyzer 2100 (Agilent). The RNA-seq analysis was performed at BGI via Illumina HiSeq 2000 platform. Briefly, mRNAs were purified by oligo (dT)-attached magnetic beads and fragmented into small pieces, followed by double-stranded cDNA synthesis. Then, the cDNA was subjected to end-repair and 3′ adenylation followed by adaptor ligation to the ends of these cDNA fragments. After that, the PCR amplification was performed for sequencing. Principal component analysis (PCA) was performed using ggord package in R. To identify known pathways concerning the differentially expressed genes (DEGs), we used online software metascape to perform the enrichment analysis.

### ChIP assay

GM-CSF- and IL4-differentiated BMDCs were used for immunoprecipitation. Cells were cross-linked on a rotator with 1% methanol-free formaldehyde for 10 minutes at room temperature. Cross-linking was quenched by adding glycine into the cross-linked samples to a final concentration of 125 mM and rotated for 5 minutes, and cell pellets were washed twice with ice-cold PBS. Subsequently, cells were lysed in sonication buffer (10 mM Tris [pH 8.0], 100 mM NaCl, 1 mM EDTA, 0.5 mM EGTA, 0.1% sodium deoxycholate, and 0.5% N-lauroylsarcosine) at 4°C and sonicated for four cycles (10 seconds on and 20 seconds off per cycle) to obtain DNA fragments with 500 bp on average. Chromatins were then immunoprecipitated overnight with 1–2 μg of respective antibodies and then incubated with Dynabeads Protein A or G (Invitrogen) for 2 hours at 4°C. Beads containing protein–DNA complexes were washed seven times with RIPA buffer (50 mM HEPES [pH 7.5], 500 mM LiCl, 1 mM EDTA, 1% NP-40, and 0.7% sodium deoxycholate), followed by washing with 1 mM Tris-EDTA/300 mM NaCl, and 1 mM Tris-EDTA/50 mM NaCl (once each). Cross-linking was reversed by boiling the samples at 100°C for 15 minutes, and DNAs were eluted with 100 μl extraction buffer (1% SDS, 100 mM NaHCO_3_, and 300 mM NaCl) by vortex. Next, proteins were degraded with 1 mg/ml proteinase K at 55°C, and DNAs were extracted by a DNA purification kit (Axygen). Both immunoprecipitated DNAs and input DNAs were resuspended in 100 μl 0.1× TE buffer for real-time PCR.

### Statistical analysis

Log-rank (Mantel–Cox) test and Student’s *t* test were used for statistical analysis; *p* < 0.05 is considered as significant.

## Supporting information

S1 Fig DC-specific *Tsc1* ablation does not affect DC development. (A) Total mRNAs extracted from spleen DCs, CD8^+^ T cells, CD4^+^ T cells, B cells, Macs, and NK cells of WT and TSC1^DC-KO^ mice were analyzed by real-time PCR. The data are presented as means ± SEM (**p < 0.01; analyzed by Student’s *t* test). (B) Whole-cell lysates were prepared from WT and TSC1^DC−/−^ splenic DCs, CD8^+^ T cells, and CD4^+^ T cells and probed with indicated antibodies. (C) The percentages and numbers of cDC subsets in the thymus of WT and TSC1^DC-KO^ mice (*n* = 3) were analyzed by flow cytometry. (D) The expression levels of CCR7 in the migratory DCs from WT and TSC1^DC-KO^ pLNs (*n* = 4) were analyzed by flow cytometry. (E) The percentages of cDCs (CD11c^+^MHC-II^+^, pregated as F4/80^−^CD64^−^) and cDC subsets (XCR1^+^ and SIRPα^+^ cDCs) in kidneys of WT and TSC1^DC-KO^ mice (*n* = 6) were analyzed by flow cytometry. The total cell numbers were counted by a hemocytometer under a microscope. (F) The percentages of total T cells (CD3^+^) and T-cell subsets (CD8^+^ and CD4^+^ T cells) of pLNs from WT and TSC1^DC-KO^ mice were analyzed by flow cytometry. (G) The percentages of total T cells (CD3^+^) and T-cell subsets (CD8^+^ and CD4^+^ T cells) and B cells (CD19^+^B220^+^) of mLNs from WT and TSC1^DC-KO^ mice were analyzed by flow cytometry. (H) Naïve and memory–phenotype CD4^+^ T cells of WT and TSC1^DC-KO^ spleens (*n* = 4) were analyzed by flow cytometry, and the percentages were calculated. The data are presented as means ± SEM (**p < 0.01; analyzed by Student’s *t* test). These experiments were repeated at least once with similar results. Underlying data are available in S1 Data and S1 Raw Images. CCR7, chemokine (C-C motif) receptor 7; CD, cluster of differentiation; cDC, classical DC; DC, dendritic cell; Mac, macrophage; MFI, mean fluorescence intensity; MHC, major histocompatibility complex; Mig DC, migratory DC; mLN, mesenteric lymph node; NK, natural killer cell; pLN, peripheral lymph node; SIRPα, signal regulatory protein α; SSC, side scatter; T_CM_, central memory T cell; T_EM_, effector memory T cell; T_N_, naïve T cell; Tsc1, tuberous sclerosis complex subunit 1; TSC1^DC-KO^, specific ablation of *Tsc1* in the DC compartment; WT, wild-type; XCR1, chemokine (C motif) receptor 1.
(TIF)

S2 Fig TSC1-mTORC1 in DCs, rather than macrophages, affects CD8 T-cell homeostasis. (A and B) The percentages of total T cells (CD3^+^) and T-cell subsets (CD8^+^ and CD4^+^ T cells) of spleens and pLNs from WT, TSC1^DC-KO^, and TSC1/mTOR^DC-DKO^ mice (A) or TSC1/Raptor^DC-DKO^ mice (B) were analyzed by flow cytometry. (C) Spleens from WT and TSC1^DC-KO^ mice (*n* = 6) were isolated and immediately stained with annexin V and PI; after cell surface marker staining, early apoptotic CD4^+^ T cells (annexin V^+^PI^−^) were calculated. The data are presented as means ± SEM (***p < 0.001, analyzed by Student’s *t* test). (D) The percentages of total T cells (CD3^+^) and T-cell subsets (CD8^+^ and CD4^+^ T cells) and B cells (CD19^+^B220^+^) of spleens, pLNs, and mLNs and percentages of different T-cell populations in thymuses from WT and TSC1^M/N-KO^ mice were analyzed by flow cytometry. The data are presented as means ± SEM. These experiments were repeated at least once, and similar results were obtained. Underlying data are available in S1 Data. CD, cluster of differentiation; DC, dendritic cell; DN, double negative; DP, double positive; mLN, mesenteric lymph node; mTor, mechanistic target of rapamycin; mTORC1, mTOR complex 1; PI, propidium iodide; pLN, peripheral lymph node; Rptor, regulatory associated protein of MTORc1; SP, single positive; SSC, side scatter; Tsc1, tuberous sclerosis complex subunit 1; TSC1^DC-KO^, specific ablation of *Tsc1* in the DC compartment; WT, wild-type.
(TIF)

S3 Fig mTOR ablation restores CD8 T-cell responses in TSC1^DC-KO^ mice. (A) The 6–8-week-old WT and TSC1^DC-KO^ littermates (*n* = 4) were i.v. infected with 10^4^ CFU of L.M-OVA. After 7 days, the spleens were isolated, and KLRG1^+^ and CD44^+^ CD8^+^ T cells were analyzed by flow cytometry, and the percentages of different type of cells among CD8^+^ T cells and cell numbers were calculated; the data are presented as means ± SEM (**p < 0.01, ***p < 0.001; analyzed by Student’s *t* test). (B) In total, 5 × 10^6^ splenocytes from infected mice were restimulated with 10 ng/ml OVA_257-264_ for 5 hours in the presence of brefeldin A. The percentages of IFNγ- and TNF-producing CD8^+^ T cells were analyzed by intracellular staining followed with flow cytometry. The data are presented as means ± SEM. These experiments were conducted three times with similar results. (C) The 6–8-week-old WT, TSC1^DC-KO^, and TSC1/mTOR^DC-DKO^ mice (*n* = 4) were infected with L.M.-OVA as in (A). After 7 days, the spleens were isolated and 5 × 10^6^ splenocytes from infected mice were restimulated with 10 ng/ml OVA_257-264_ for 5 hours in the presence of brefeldin A. The percentages of IFNγ-producing CD8^+^ T cells were analyzed by intracellular staining followed by flow cytometry, and cell numbers were calculated accordingly (left panel). The percentages and numbers of the OVA-specific CD8^+^ T cells were also analyzed by flow cytometry (right panel). The data are presented as means ± SEM (*p < 0.05, **p < 0.01, ***p < 0.001; analyzed by Student’s *t* test). This experiment was performed twice with similar results. (D) WT, TSC1^DC-KO^, and TSC1/mTOR^DC-DKO^ mice (*n* = 6) were injected s.c. with 5 × 10^5^ B16-OVA melanoma cells, and the tumor size was measured every 2 days. This experiment was repeated once with similar results. The data are shown as means ± SEM (**p < 0.01, ***p < 0.001, analyzed by Student’s *t* test). Underlying data are available in S1 Data. CD, cluster of differentiation; CFU, colony-forming unit; DC, dendritic cell; IFN, interferon; i.v., intravenously; KLRG1, killer cell lectin-like receptor subfamily G, member 1; L.M., *L*. *monocytogenes*; OVA, ovalbumin; mTOR, mechanistic target of rapamycin; s.c., subcutaneously; TNF, tumor necrosis factor; Tsc1, tuberous sclerosis complex subunit 1; TSC1^DC-KO^, specific ablation of *Tsc1* in the DC compartment; WT, wild-type.
(TIF)

S4 Fig TSC1-deficient DCs express normal cytokines/costimulatory molecules upon activation. (A) BMDCs were seeded into 96-well plates (10^5^ cells per well) and then either NT or treated with 100 ng/ml LPS overnight (LPS). Secreted IL-6, IL-12p40, TNF, IL-10, CXCL-1, and CXCL-10 in the supernatants were quantified by ELISA. The data are shown as means ± SEM. (B) WT and TSC1^DC-KO^ BMDCs were either NT or treated with 100 ng/ml of LPS overnight (LPS). The expression levels of CD80, CD86, CD40, H2-K^b^, and MHC-II were analyzed by flow cytometry. (C and D) The expression levels of CCR7, CXCR4, and CD62L in CD8^+^ T and CD4^+^ T cells (C) and IL7Rα in CD4^+^ T cells (D) from WT and TSC1^DC-KO^ spleens (*n* = 4) were analyzed by flow cytometry. The data are presented as means ± SEM (*p < 0.05, ***p < 0.001; analyzed by Student’s *t* test). (E) Total mRNAs were extracted from the CD4^+^ T cells of WT and TSC1^DC-KO^ spleens, and *Il2* expression was measured by real-time PCR. The data are presented as means ± SEM. These experiments were repeated once with similar results. Underlying data are available in S1 Data. BMDC, bone marrow–derived DC; CCR7, chemokine (C-C motif) receptor 7; CD, cluster of differentiation; Ctrl, control; CXCL, chemokine (C-X-C motif) ligand; CXCR4, chemokine (C-X-C motif) receptor 4; DC, dendritic cell; H2-K^b^, histocompatibility 2, K1, K region; IL, interleukin; LPS, lipopolysaccharide; MFI, mean fluorescence intensity; MHC, major histocompatibility complex; NT, untreated; TNF, tumor necrosis factor; Tsc1, tuberous sclerosis complex subunit 1; TSC1^DC-KO^, specific ablation of *Tsc1* in the DC compartment; WT, wild-type.
(TIF)

S5 Fig Analyses of lipids synthesis, FAO, and glutaminolysis. (A) Principal component analysis of differential expression genes between WT and TSC1^DC-KO^ groups. (B) Top 14 signaling pathways involved by differentially expressed genes (log [q value] < −5, number of hit genes > 20). (C) BMDCs were incubated with cell culture medium containing 100 nM mitotracker for 30 minutes at 37°C, and the fluorescent intensity was measured by flow cytometry. (D) OCR was analyzed in TSC1^DC-KO^ BMDCs by a Seahorse analyzer following sequential treatment with 1 μM oligomycin, 1.5 μM FCCP, 200 μM Eto/100 mM 2-DG/10 μM BPTES, and 100 nM rotenone plus 1 μM antimycin A. OCR after treatment with FCCP was set as 100%. The data are shown as means ± SEM. (E) Cell surface and intracellular MHC-I (H2-K^b^) and MHC-II expression levels of different splenic DC subsets (CD8^+^ DCs and CD11b^+^ DCs) from WT and TSC1^DC-KO^ mice were analyzed by flow cytometry. Underlying data are available in S1 Data. 2-DG, 2-deoxy-D-glucose; BMDC, bone marrow–derived DC; BPTES, bis-2-(5-phenylacetamido-1, 3, 4-thiadiazol-2-yl) ethyl sulfide; CD, cluster of differentiation; Ctrl, control; DC, dendritic cell; Eto, etomoxir; FAO, fatty acid oxidation; FCCP, fluoro-carbonyl cyanide phenylhydrazone; H2-K^b^, histocompatibility 2, K1, K region; MHC, major histocompatibility complex; OCR, oxygen consumption rate; Tsc1, tuberous sclerosis complex subunit 1; TSC1^DC-KO^, specific ablation of *Tsc1* in the DC compartment; WT, wild-type.
(TIF)

S6 Fig DC presentation of MHC-II/Ag and CD4 T-cell proliferation. (A) BMDCs were either NT or stimulated with 100 ng/ml of LPS overnight (LPS) and then pulsed with 100 ng/ml of OVA_257-264_ for 1 hour. The cell surface H2-K^b^/OVA_257-264_ complexes were analyzed by flow cytometry. (B) BMDCs were incubated with 1 mg/ml of TMR-Dextran at 37°C for indicated times, and phagocytosis was measured by flow cytometry. (C) BMDCs and spleen DCs pulsed with 1 μg/ml of OVA_323-339_ or 0.25 mg/ml OVA for 1 hour and 6 hours, respectively, were cocultured with CFSE-labeled OT-II CD4^+^ T cells for 3 days. The proliferation of OT-II CD4^+^ T cells were analyzed by division of CFSE by flow cytometry. (D) Control and H2-K^b^ expressing lentiviruses vectors were transfected into BMDCs, and H2Kb levels were analyzed by flow cytometry. These experiments were repeated at least once with similar results. Underlying data are available in S1 Data. Ag, antigen; BMDC, bone marrow–derived DC; CFSE, carboxyfluorescein diacetate succinimidyl ester; Ctrl, control; DC, dendritic cell; GFP, green fluorescent protein; H2-K^b^, histocompatibility 2, K1, K region; LPS, lipopolysaccharide; MHC, major histocompatibility complex; MFI, mean fluorescence intensity; NT, untreated; OT, ovalbumin-specific TCR transgenic mouse; OVA, ovalbumin; TMR, tetramethylrhodamine; Tsc1, tuberous sclerosis complex subunit 1; TSC1^DC-KO^, specific ablation of *Tsc1* in the DC compartment; WT, wild-type.
(TIF)

S7 Fig TOFA, but not C75 or 2-DG, elevates DC-mediated OT-I proliferation. (A) Whole-cell lysates were prepared from WT and TSC1^DC-KO^ splenic DCs, and immunoblotting was performed with indicated antibodies. This experiment was repeated twice with similar results, and the representative data were shown. (B) BMDCs were either untreated or treated with indicated inhibitors for 24 hours, and total mRNAs were extracted. The expression levels of *H2-Aa* and *H2-Ab1* were measured by real-time PCR. The data are presented as means ± SEM. (C) BMDCs were either untreated or treated with 1 mM 2-DG for 24 hours and were pulsed with 0.1 ng/ml OVA_257-264_ (left) or 0.25 mg/ml OVA (right) for 1 hour or 6 hours, respectively, then cocultured with CFSE-labeled OT-I CD8^+^ T cells for 2 days. The proliferation of OT-I CD8^+^ T cells were analyzed by division of CFSE by flow cytometry. The proliferation percentages were analyzed. The data are presented as means ± SEM. (D) TSC1^−/−^ BMDCs were either untreated or treated with 15 μM ACLYi (BMS-303141) for 24 hours, and the expression levels of H2-K^b^ and H2-D^b^ were analyzed by flow cytometry. (E) WT and TSC1^−/−^ BMDCs were either untreated or treated with 20 μM TOFA or 5 μM C75 for 24 hours, pulsed with 0.1 ng/ml OVA_257-264_ for 1 hour, and then cocultured with CFSE-labeled purified OT-I CD8^+^ T cells for 2 days. The proliferation of OT-I CD8^+^ T cells were analyzed by division of CFSE by flow cytometry. The data are presented as means ± SEM (*p < 0.05, analyzed by Student’s *t* test). These experiments were repeated at least once. Underlying data are available in S1 Data and S1 Raw Images. 2-DG, 2-deoxy-D-glucose; 25-HC, 25-hydroxycholesterol; ACLY, ATP-citrate lyase; ACLYi, ACLY inhibitor; BMDC, bone marrow–derived DC; CFSE, carboxyfluorescein diacetate succinimidyl ester; CREB, cAMP responsive element binding protein; Ctrl, control; DC, dendritic cell; H2-Aa, histocompatibility 2, class II antigen A, alpha; H2-Ab1, histocompatibility 2, class II antigen A, beta 1; H2-D^b^, histocompatibility 2, D region locus 1; H2-K^b^, histocompatibility 2, K1, K region; IκBα, NF-kappa-B inhibitor alpha; IKK, inhibitor of kappaB kinase; OT, ovalbumin-specific TCR transgenic mouse; OVA, ovalbumin; TOFA, 5-(tetradecyloxy)-2-furoic acid; Tsc1, tuberous sclerosis complex subunit 1; TSC1^DC-KO^, specific ablation of *Tsc1* in the DC compartment; WT, wild-type.
(TIF)

S1 Table Primers used in this study. (XLSX)

S1 Data Numerical values of presented diagrams. (XLSX)

S1 Raw Images Uncropped western blots data. (PDF)

## Abbreviations

2-DG: 2-deoxy-D-glucose
2-NBDG: 2-deoxy-2-[(7-nitro-2,1,3-benzoxadiazol-4-yl)amino]-D-glucose
α-KG: α-ketoglutarate
Acaca: acetyl-CoA carboxylase alpha
ACC1: acetyl-CoA carboxylase 1
acetyl-CoA: acetyl-coenzyme A
Acly: ATP-citrate lyase
Aclyi: Acly inhibitor
ACSS2: acyl-CoA synthetase short chain family member 2
AMPK: AMP-activated protein kinase
B2m: beta-2 microglobulin
BMDC: bone marrow–derived DC
BODIPY: boron-dipyrromethene
BPTES: bis-2-(5-phenylacetamido-1, 3, 4-thiadiazol-2-yl) ethyl sulfide
BrdU: bromodeoxyuridine
CCR7: chemokine (C-C motif) receptor 7
CD: cluster of differentiation
cDC: classical DC
c-Fos: FBJ osteosarcoma oncogene
CFSE: carboxyfluorescein diacetate succinimidyl ester
ChIP: chromatin immunoprecipitation
CLR: C-type lectin receptor
c-Myc: cellular myelocytomatosis
Cre: cyclization recombination enzyme
CREB: cAMP responsive element binding protein
CreER: Cre-estrogen receptor
CXCL: chemokine (C-X-C motif) ligand
CXCR4: chemokine (C-X-C motif) receptor 4
DAMP: danger-associated molecule pattern
DC: dendritic cell
DTA: diphtheria toxin A
ECAR: extracellular acidification rate
ER: endoplasmic reticulum
FAO: fatty acid oxidation
Fasn: fatty acid synthase
FRC: fibroblast reticular cell
Glut1: glucose transporter 1
H2-Aa: histocompatibility 2, class II antigen A, alpha
H2-Ab1: histocompatibility 2, class II antigen A, beta 1
H2-D1: histocompatibility 2, D region locus 1
H2-D^b^: histocompatibility 2, D region locus 1
H2-K1: histocompatibility 2, K1, K region
H2-K^b^: histocompatibility 2, K1, K region
H3K27ac: acetyl-histone H3 (Lys27)
H3K27me3: tri-methyl-histone H3 (Lys27)
H3K9ac: acetyl-histone H3 (Lys9)
H3K9me3: tri-methyl-histone H3 (Lys9)
HIF-1α: hypoxia-inducible factor-1α
Hk2: hexokinase 2
Hmgcr: 3-hydroxy-3-methylglutaryl-coenzyme A reductase
IFN: interferon
IgG: immunoglobulin G
IKK: inhibitor of kappaB kinase
IL: interleukin
IRF: IFN regulatory factor
KLRG1: killer cell lectin-like receptor subfamily G, member 1
LamTOR: late endosomal/lysosomal adaptor, MAPK and MTOR activator
Ldha: lactate dehydrogenase A
L.M.: *Listeria monocytogenes*
LN: lymph node
LPS: lipopolysaccharide
Lyz2: lysozyme 2
M2 macrophage: alternatively activated macrophage
malonyl-CoA: malonyl-coenzyme A
MFI: mean fluorescence intensity
MHC: major histocompatibility complex
Mst1/2: mammalian sterile-20-like ½
mTOR: mechanistic target of rapamycin
mTORC1: mTOR complex 1
mTORC2: mTOR complex 2
NaAc: sodium acetate
NCBI: National Center for Biotechnology Information
NFκB: nuclear factor kappa B
NK: natural killer cell
OCR: oxygen consumption rate
OT: ovalbumin-specific TCR transgenic mouse
OVA: ovalbumin
OXPHOS: oxidative phosphorylation
PAMP: pathogen-associated molecule pattern
pDC: plasmacytoid DC
PDCA1: pDC Ag-1
PI: propidium iodide
pLN: peripheral lymph node
PRR: pattern-recognition receptor
PTEN: phosphatase and tensin homolog
RNA-seq: RNA sequencing
Rptor: regulatory associated protein of MTORc1
SAM: S-adenosylmethionine
Scap: sterol regulatory element-binding transcription factor chaperone
shRNA: short hairpin RNA
SIRPα: signal regulatory protein α
Slc2a1: solute carrier family 2 member 1
SRC: spare respiratory capacity
SSC: side scatter
TCA: tricarboxylic acid
TCR: T-cell receptor
Th2: T helper 2
TLR: Toll-like receptor
TNF: tumor necrosis factor
TOFA: 5-(tetradecyloxy)-2-furoic acid
TSC1: tuberous sclerosis complex subunit 1
TSC1^DC-KO^: specific ablation of *Tsc1* in the DC compartment
TSC2: tuberous sclerosis complex subunit 2
XCR1: chemokine (C motif) receptor 1
