# Supplementary figures and images for: TSC1/mTOR-controlled metabolic–epigenetic cross talk underpins DC control of CD8+ T-cell homeostasis
Source: PLoS Biol. 2019 Aug 21;17(8):e3000420. doi: 10.1371/journal.pbio.3000420 (PMC6719877; doi:10.1371/journal.pbio.3000420)

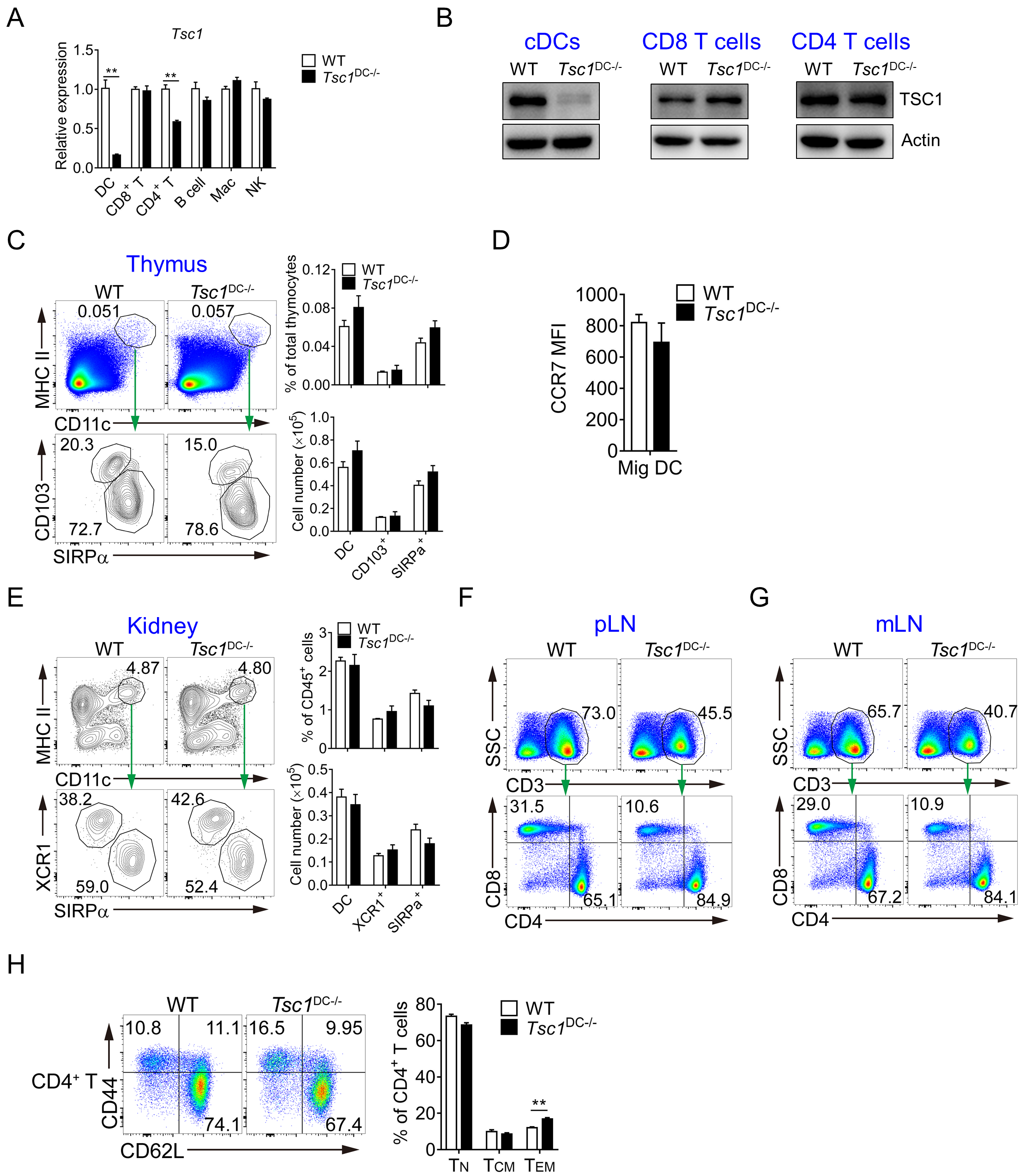

Supplement: S1 Fig — (A) Total mRNAs extracted from spleen DCs, CD8+ T cells, CD4+ T cells, B cells, Macs, and NK cells of WT and TSC1DC-KO mice were analyzed by real-time PCR. The data are presented as means ± SEM (**p < 0.01; analyzed by Student’s t test). (B) Whole-cell lysates were prepared from WT and TSC1DC−/− splenic DCs, CD8+ T cells, and CD4+ T cells and probed with indicated antibodies. (C) The percentages and numbers of cDC subsets in the thymus of WT and TSC1DC-KO mice (n = 3) were analyzed by flow cytometry. (D) The expression levels of CCR7 in the migratory DCs from WT and TSC1DC-KO pLNs (n = 4) were analyzed by flow cytometry. (E) The percentages of cDCs (CD11c+MHC-II+, pregated as F4/80−CD64−) and cDC subsets (XCR1+ and SIRPα+ cDCs) in kidneys of WT and TSC1DC-KO mice (n = 6) were analyzed by flow cytometry. The total cell numbers were counted by a hemocytometer under a microscope. (F) The percentages of total T cells (CD3+) and T-cell subsets (CD8+ and CD4+ T cells) of pLNs from WT and TSC1DC-KO mice were analyzed by flow cytometry. (G) The percentages of total T cells (CD3+) and T-cell subsets (CD8+ and CD4+ T cells) and B cells (CD19+B220+) of mLNs from WT and TSC1DC-KO mice were analyzed by flow cytometry. (H) Naïve and memory–phenotype CD4+ T cells of WT and TSC1DC-KO spleens (n = 4) were analyzed by flow cytometry, and the percentages were calculated. The data are presented as means ± SEM (**p < 0.01; analyzed by Student’s t test). These experiments were repeated at least once with similar results. Underlying data are available in S1 Data and S1 Raw Images. CCR7, chemokine (C-C motif) receptor 7; CD, cluster of differentiation; cDC, classical DC; DC, dendritic cell; Mac, macrophage; MFI, mean fluorescence intensity; MHC, major histocompatibility complex; Mig DC, migratory DC; mLN, mesenteric lymph node; NK, natural killer cell; pLN, peripheral lymph node; SIRPα, signal regulatory protein α; SSC, side scatter; TCM, central memory T cell; TEM, effector memory T cell; [file pbio.3000420.s001.TIF]

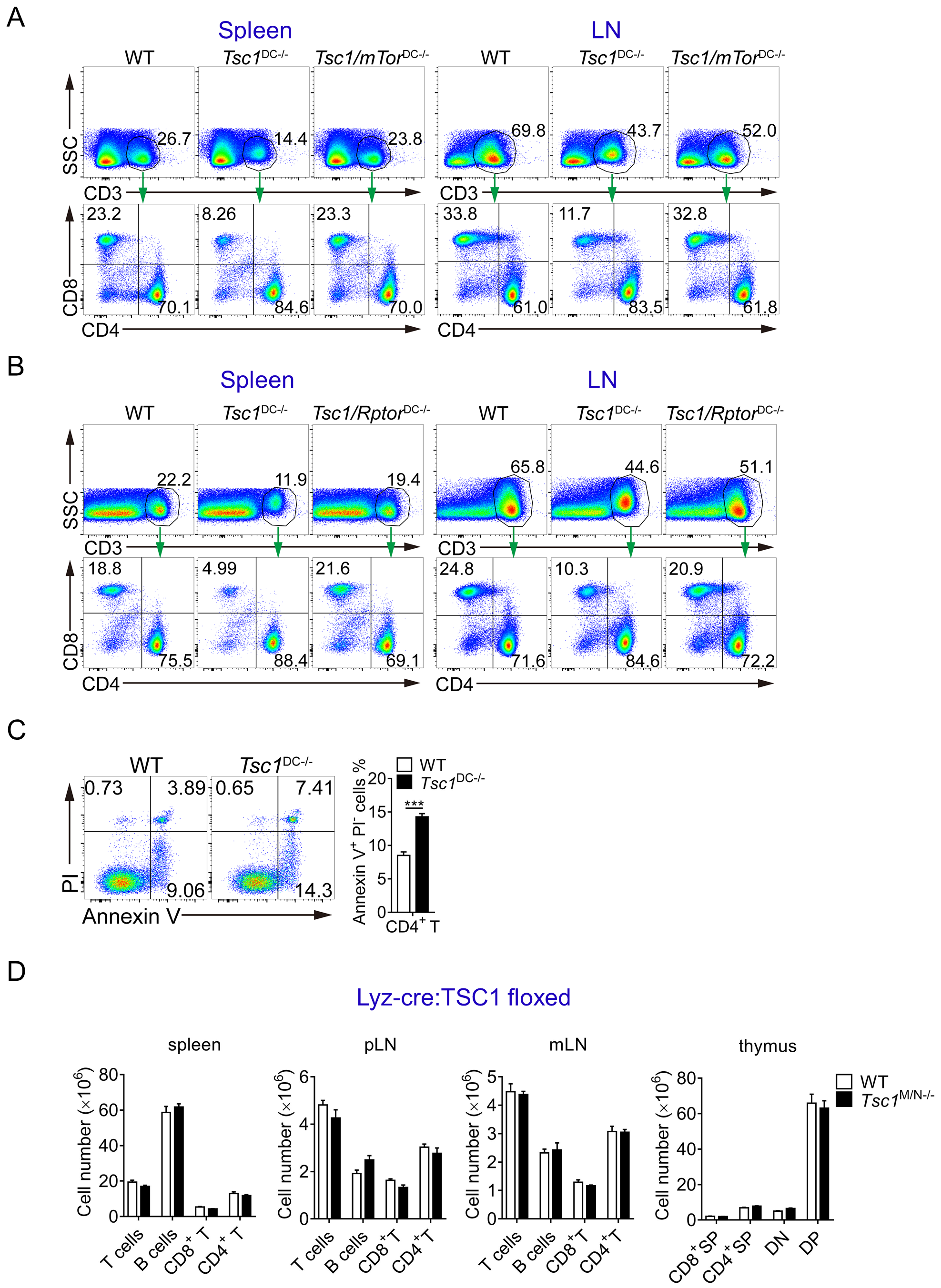

Supplement: S2 Fig — (A and B) The percentages of total T cells (CD3+) and T-cell subsets (CD8+ and CD4+ T cells) of spleens and pLNs from WT, TSC1DC-KO, and TSC1/mTORDC-DKO mice (A) or TSC1/RaptorDC-DKO mice (B) were analyzed by flow cytometry. (C) Spleens from WT and TSC1DC-KO mice (n = 6) were isolated and immediately stained with annexin V and PI; after cell surface marker staining, early apoptotic CD4+ T cells (annexin V+PI−) were calculated. The data are presented as means ± SEM (***p < 0.001, analyzed by Student’s t test). (D) The percentages of total T cells (CD3+) and T-cell subsets (CD8+ and CD4+ T cells) and B cells (CD19+B220+) of spleens, pLNs, and mLNs and percentages of different T-cell populations in thymuses from WT and TSC1M/N-KO mice were analyzed by flow cytometry. The data are presented as means ± SEM. These experiments were repeated at least once, and similar results were obtained. Underlying data are available in S1 Data. CD, cluster of differentiation; DC, dendritic cell; DN, double negative; DP, double positive; mLN, mesenteric lymph node; mTor, mechanistic target of rapamycin; mTORC1, mTOR complex 1; PI, propidium iodide; pLN, peripheral lymph node; Rptor, regulatory associated protein of MTORc1; SP, single positive; SSC, side scatter; Tsc1, tuberous sclerosis complex subunit 1; TSC1DC-KO, specific ablation of Tsc1 in the DC compartment; WT, wild-type. (TIF) [file pbio.3000420.s002.TIF]

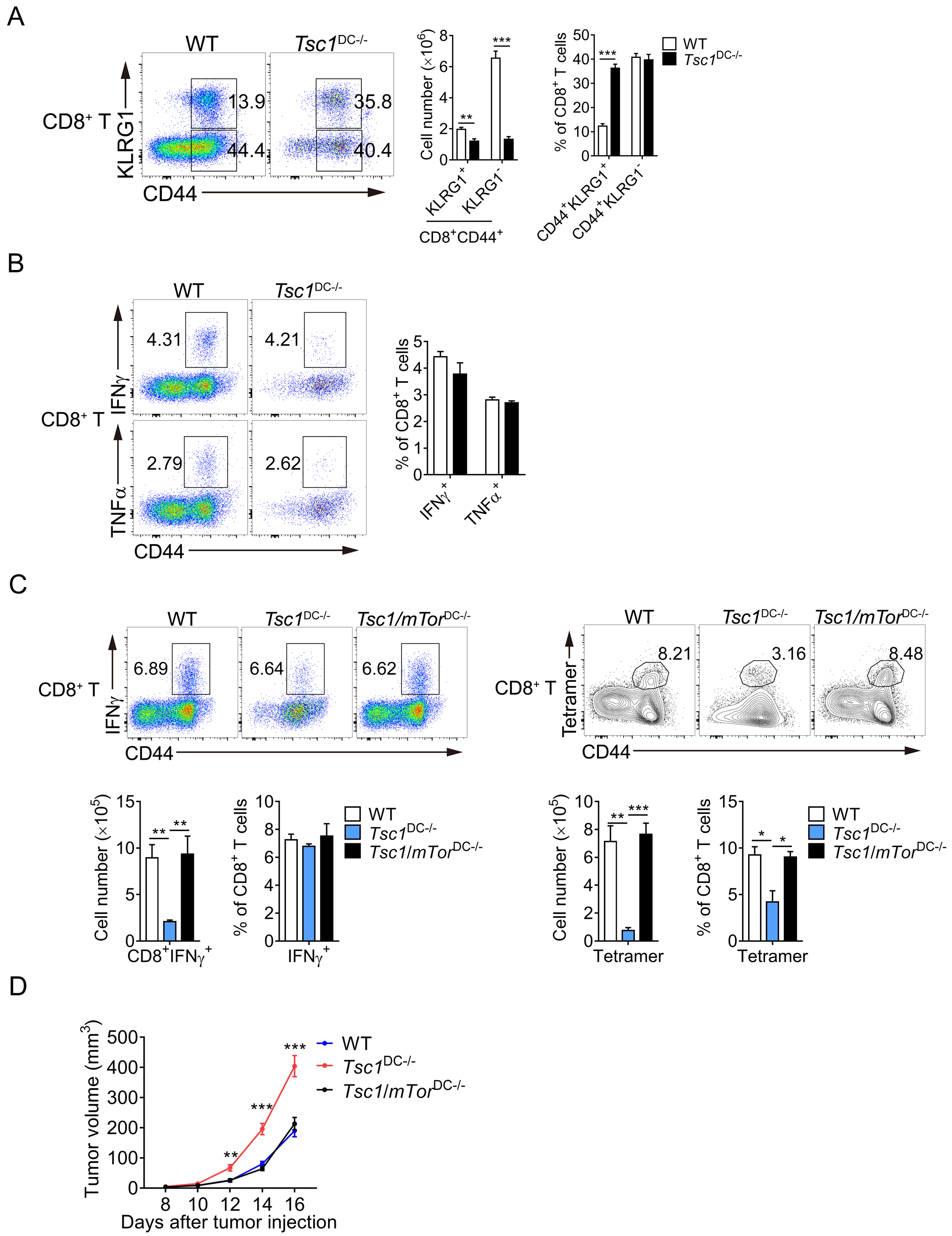

Supplement: S3 Fig — (A) The 6–8-week-old WT and TSC1DC-KO littermates (n = 4) were i.v. infected with 104 CFU of L.M-OVA. After 7 days, the spleens were isolated, and KLRG1+ and CD44+ CD8+ T cells were analyzed by flow cytometry, and the percentages of different type of cells among CD8+ T cells and cell numbers were calculated; the data are presented as means ± SEM (**p < 0.01, ***p < 0.001; analyzed by Student’s t test). (B) In total, 5 × 10^6^ splenocytes from infected mice were restimulated with 10 ng/ml OVA257-264 for 5 hours in the presence of brefeldin A. The percentages of IFNγ- and TNF-producing CD8+ T cells were analyzed by intracellular staining followed with flow cytometry. The data are presented as means ± SEM. These experiments were conducted three times with similar results. (C) The 6–8-week-old WT, TSC1DC-KO, and TSC1/mTORDC-DKO mice (n = 4) were infected with L.M.-OVA as in (A). After 7 days, the spleens were isolated and 5 × 10^6^ splenocytes from infected mice were restimulated with 10 ng/ml OVA257-264 for 5 hours in the presence of brefeldin A. The percentages of IFNγ-producing CD8+ T cells were analyzed by intracellular staining followed by flow cytometry, and cell numbers were calculated accordingly (left panel). The percentages and numbers of the OVA-specific CD8+ T cells were also analyzed by flow cytometry (right panel). The data are presented as means ± SEM (*p < 0.05, **p < 0.01, ***p < 0.001; analyzed by Student’s t test). This experiment was performed twice with similar results. (D) WT, TSC1DC-KO, and TSC1/mTORDC-DKO mice (n = 6) were injected s.c. with 5 × 10^5^ B16-OVA melanoma cells, and the tumor size was measured every 2 days. This experiment was repeated once with similar results. The data are shown as means ± SEM (**p < 0.01, ***p < 0.001, analyzed by Student’s t test). Underlying data are available in S1 Data. CD, cluster of differentiation; CFU, colony-forming unit; DC, dendritic cell; IFN, interferon; i.v., intravenously; KLRG1, killer cell lectin-like r [file pbio.3000420.s003.TIF]

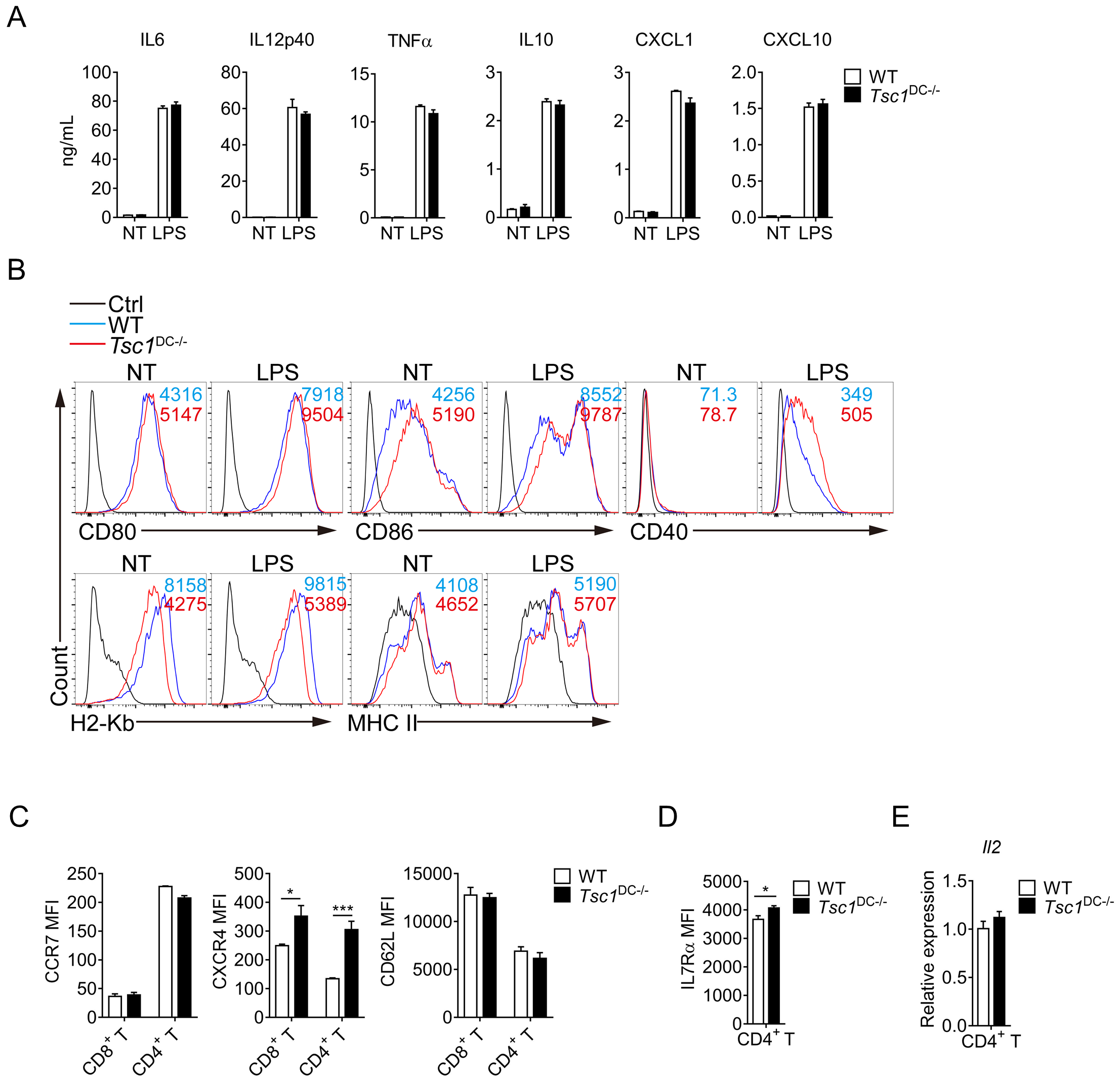

Supplement: S4 Fig — (A) BMDCs were seeded into 96-well plates (105 cells per well) and then either NT or treated with 100 ng/ml LPS overnight (LPS). Secreted IL-6, IL-12p40, TNF, IL-10, CXCL-1, and CXCL-10 in the supernatants were quantified by ELISA. The data are shown as means ± SEM. (B) WT and TSC1DC-KO BMDCs were either NT or treated with 100 ng/ml of LPS overnight (LPS). The expression levels of CD80, CD86, CD40, H2-Kb, and MHC-II were analyzed by flow cytometry. (C and D) The expression levels of CCR7, CXCR4, and CD62L in CD8+ T and CD4+ T cells (C) and IL7Rα in CD4+ T cells (D) from WT and TSC1DC-KO spleens (n = 4) were analyzed by flow cytometry. The data are presented as means ± SEM (*p < 0.05, ***p < 0.001; analyzed by Student’s t test). (E) Total mRNAs were extracted from the CD4+ T cells of WT and TSC1DC-KO spleens, and Il2 expression was measured by real-time PCR. The data are presented as means ± SEM. These experiments were repeated once with similar results. Underlying data are available in S1 Data. BMDC, bone marrow–derived DC; CCR7, chemokine (C-C motif) receptor 7; CD, cluster of differentiation; Ctrl, control; CXCL, chemokine (C-X-C motif) ligand; CXCR4, chemokine (C-X-C motif) receptor 4; DC, dendritic cell; H2-Kb, histocompatibility 2, K1, K region; IL, interleukin; LPS, lipopolysaccharide; MFI, mean fluorescence intensity; MHC, major histocompatibility complex; NT, untreated; TNF, tumor necrosis factor; Tsc1, tuberous sclerosis complex subunit 1; TSC1DC-KO, specific ablation of Tsc1 in the DC compartment; WT, wild-type. (TIF) [file pbio.3000420.s004.TIF]

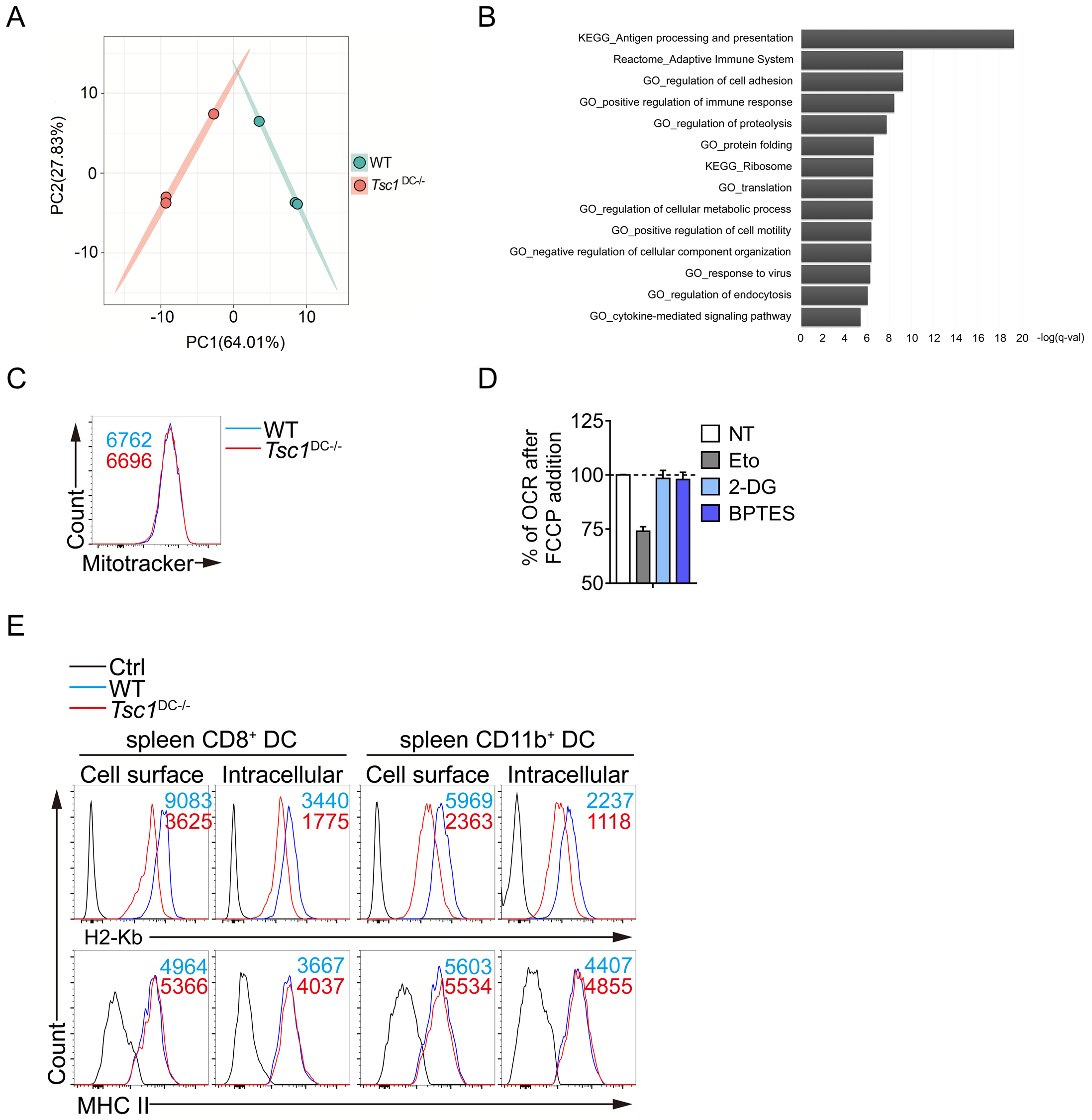

Supplement: S5 Fig — (A) Principal component analysis of differential expression genes between WT and TSC1DC-KO groups. (B) Top 14 signaling pathways involved by differentially expressed genes (log [q value] < −5, number of hit genes > 20). (C) BMDCs were incubated with cell culture medium containing 100 nM mitotracker for 30 minutes at 37°C, and the fluorescent intensity was measured by flow cytometry. (D) OCR was analyzed in TSC1DC-KO BMDCs by a Seahorse analyzer following sequential treatment with 1 μM oligomycin, 1.5 μM FCCP, 200 μM Eto/100 mM 2-DG/10 μM BPTES, and 100 nM rotenone plus 1 μM antimycin A. OCR after treatment with FCCP was set as 100%. The data are shown as means ± SEM. (E) Cell surface and intracellular MHC-I (H2-Kb) and MHC-II expression levels of different splenic DC subsets (CD8+ DCs and CD11b+ DCs) from WT and TSC1DC-KO mice were analyzed by flow cytometry. Underlying data are available in S1 Data. 2-DG, 2-deoxy-D-glucose; BMDC, bone marrow–derived DC; BPTES, bis-2-(5-phenylacetamido-1, 3, 4-thiadiazol-2-yl) ethyl sulfide; CD, cluster of differentiation; Ctrl, control; DC, dendritic cell; Eto, etomoxir; FAO, fatty acid oxidation; FCCP, fluoro-carbonyl cyanide phenylhydrazone; H2-Kb, histocompatibility 2, K1, K region; MHC, major histocompatibility complex; OCR, oxygen consumption rate; Tsc1, tuberous sclerosis complex subunit 1; TSC1DC-KO, specific ablation of Tsc1 in the DC compartment; WT, wild-type. (TIF) [file pbio.3000420.s005.TIF]

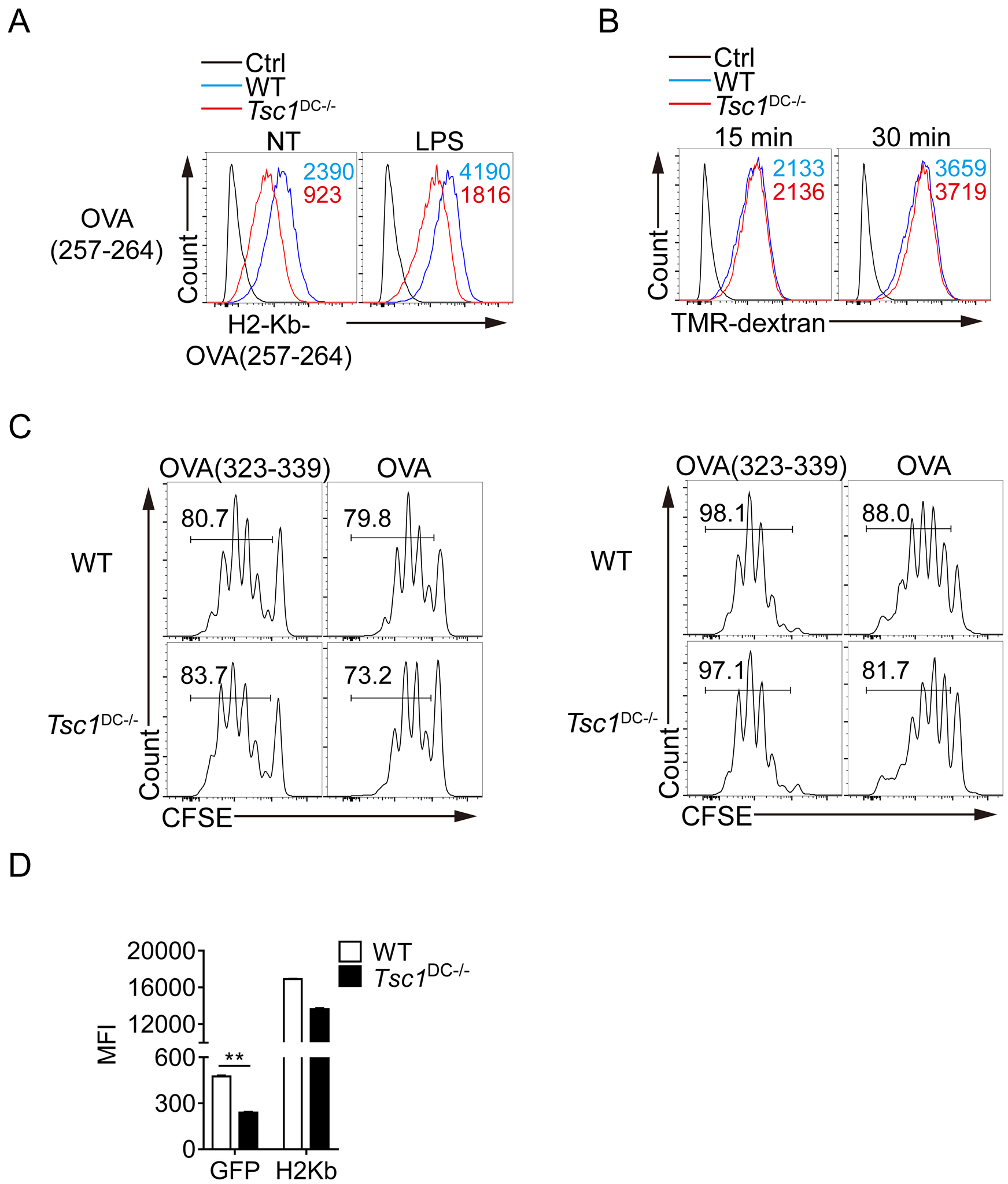

Supplement: S6 Fig — (A) BMDCs were either NT or stimulated with 100 ng/ml of LPS overnight (LPS) and then pulsed with 100 ng/ml of OVA257-264 for 1 hour. The cell surface H2-Kb/OVA257-264 complexes were analyzed by flow cytometry. (B) BMDCs were incubated with 1 mg/ml of TMR-Dextran at 37°C for indicated times, and phagocytosis was measured by flow cytometry. (C) BMDCs and spleen DCs pulsed with 1 μg/ml of OVA323-339 or 0.25 mg/ml OVA for 1 hour and 6 hours, respectively, were cocultured with CFSE-labeled OT-II CD4+ T cells for 3 days. The proliferation of OT-II CD4+ T cells were analyzed by division of CFSE by flow cytometry. (D) Control and H2-Kb expressing lentiviruses vectors were transfected into BMDCs, and H2Kb levels were analyzed by flow cytometry. These experiments were repeated at least once with similar results. Underlying data are available in S1 Data. Ag, antigen; BMDC, bone marrow–derived DC; CFSE, carboxyfluorescein diacetate succinimidyl ester; Ctrl, control; DC, dendritic cell; GFP, green fluorescent protein; H2-Kb, histocompatibility 2, K1, K region; LPS, lipopolysaccharide; MHC, major histocompatibility complex; MFI, mean fluorescence intensity; NT, untreated; OT, ovalbumin-specific TCR transgenic mouse; OVA, ovalbumin; TMR, tetramethylrhodamine; Tsc1, tuberous sclerosis complex subunit 1; TSC1DC-KO, specific ablation of Tsc1 in the DC compartment; WT, wild-type. (TIF) [file pbio.3000420.s006.TIF]

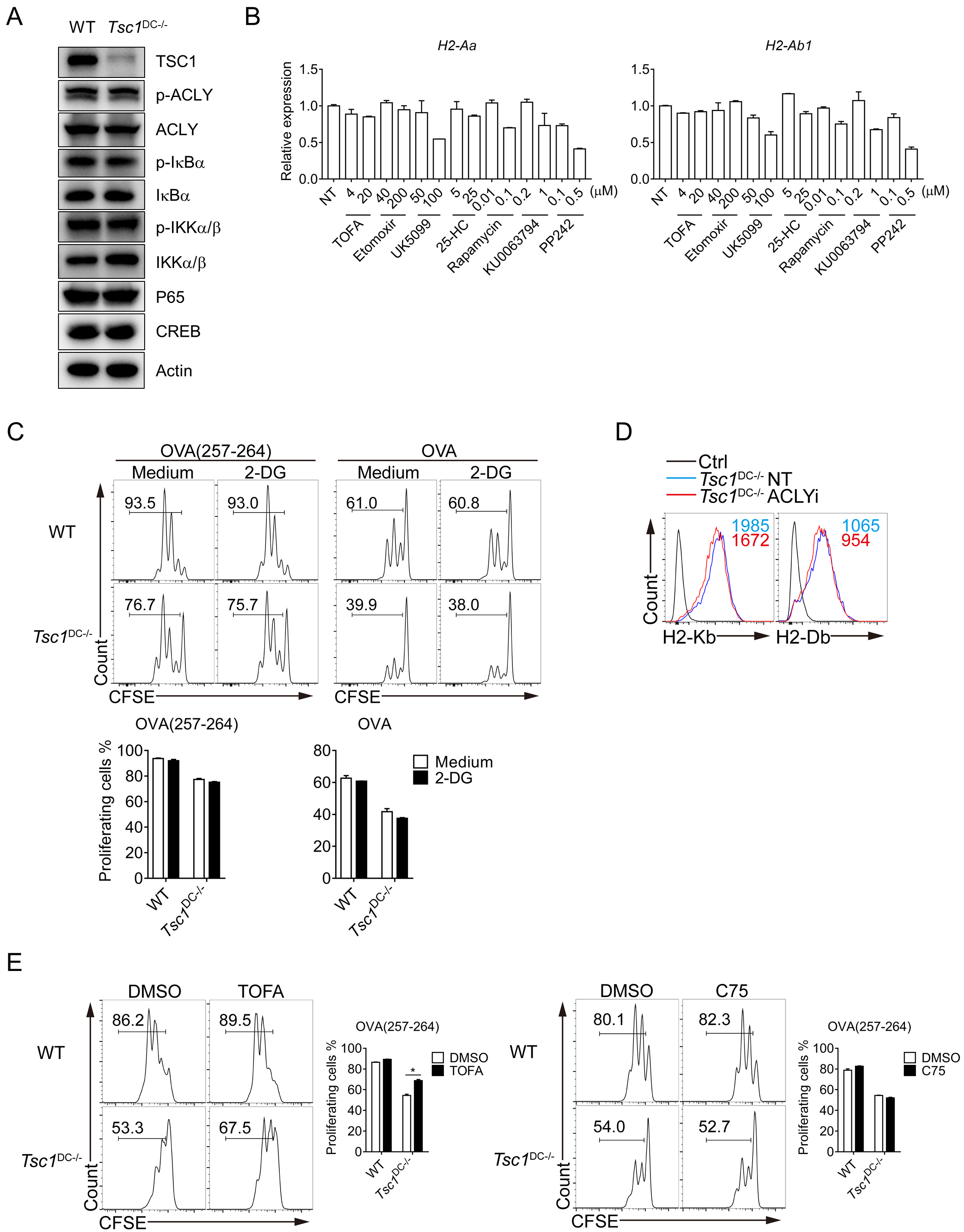

Supplement: S7 Fig — (A) Whole-cell lysates were prepared from WT and TSC1DC-KO splenic DCs, and immunoblotting was performed with indicated antibodies. This experiment was repeated twice with similar results, and the representative data were shown. (B) BMDCs were either untreated or treated with indicated inhibitors for 24 hours, and total mRNAs were extracted. The expression levels of H2-Aa and H2-Ab1 were measured by real-time PCR. The data are presented as means ± SEM. (C) BMDCs were either untreated or treated with 1 mM 2-DG for 24 hours and were pulsed with 0.1 ng/ml OVA257-264 (left) or 0.25 mg/ml OVA (right) for 1 hour or 6 hours, respectively, then cocultured with CFSE-labeled OT-I CD8+ T cells for 2 days. The proliferation of OT-I CD8+ T cells were analyzed by division of CFSE by flow cytometry. The proliferation percentages were analyzed. The data are presented as means ± SEM. (D) TSC1−/− BMDCs were either untreated or treated with 15 μM ACLYi (BMS-303141) for 24 hours, and the expression levels of H2-Kb and H2-Db were analyzed by flow cytometry. (E) WT and TSC1−/− BMDCs were either untreated or treated with 20 μM TOFA or 5 μM C75 for 24 hours, pulsed with 0.1 ng/ml OVA257-264 for 1 hour, and then cocultured with CFSE-labeled purified OT-I CD8+ T cells for 2 days. The proliferation of OT-I CD8+ T cells were analyzed by division of CFSE by flow cytometry. The data are presented as means ± SEM (*p < 0.05, analyzed by Student’s t test). These experiments were repeated at least once. Underlying data are available in S1 Data and S1 Raw Images. 2-DG, 2-deoxy-D-glucose; 25-HC, 25-hydroxycholesterol; ACLY, ATP-citrate lyase; ACLYi, ACLY inhibitor; BMDC, bone marrow–derived DC; CFSE, carboxyfluorescein diacetate succinimidyl ester; CREB, cAMP responsive element binding protein; Ctrl, control; DC, dendritic cell; H2-Aa, histocompatibility 2, class II antigen A, alpha; H2-Ab1, histocompatibility 2, class II antigen A, beta 1; H2-Db, histocompatibility 2, D region locus 1; H2-Kb, histoco [file pbio.3000420.s007.TIF]

Fig 6A

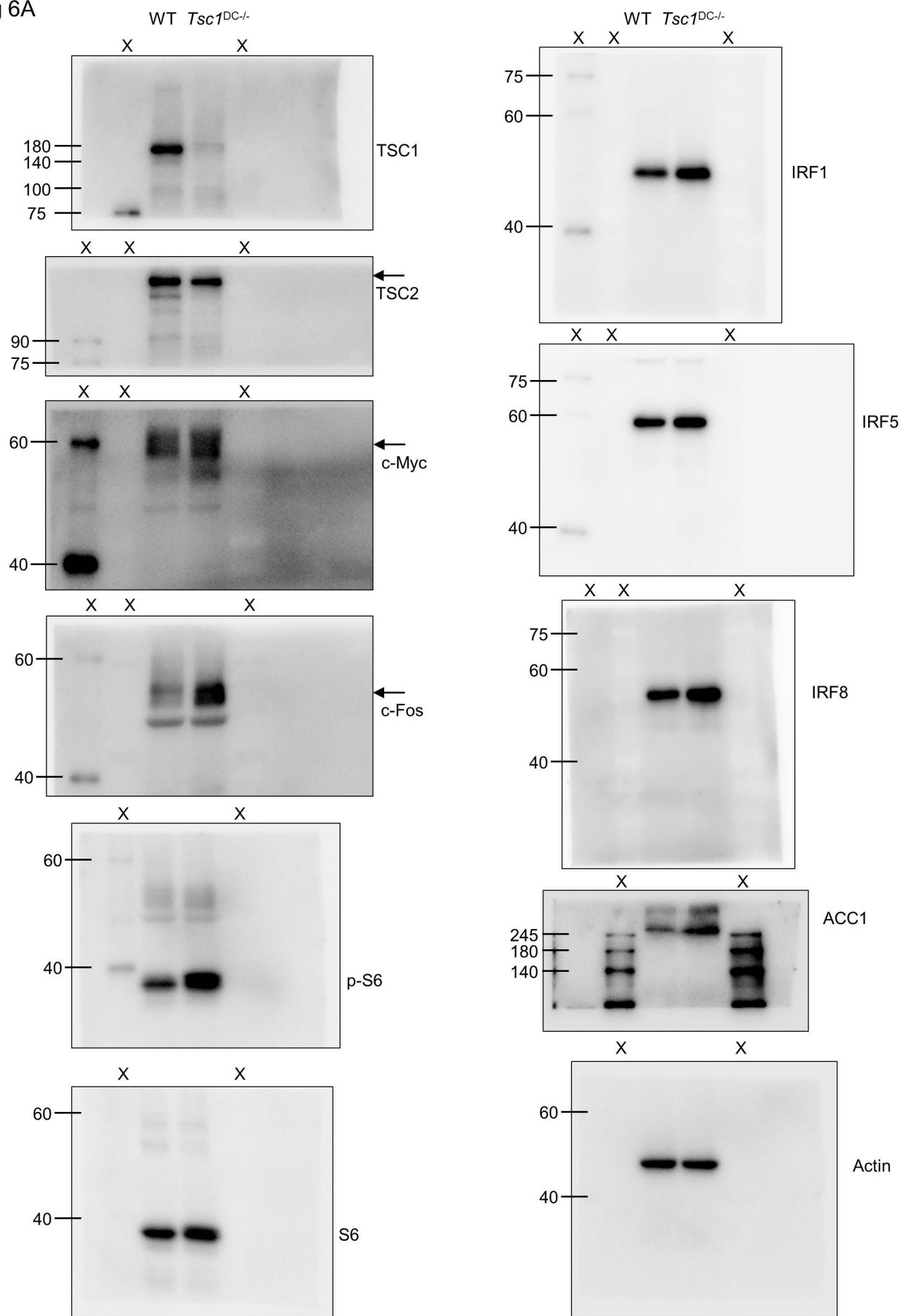

Fig 7A

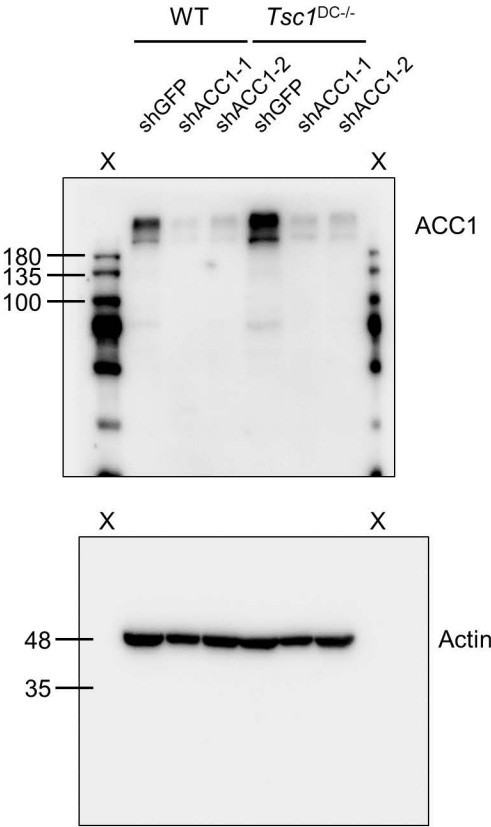

Fig 7G

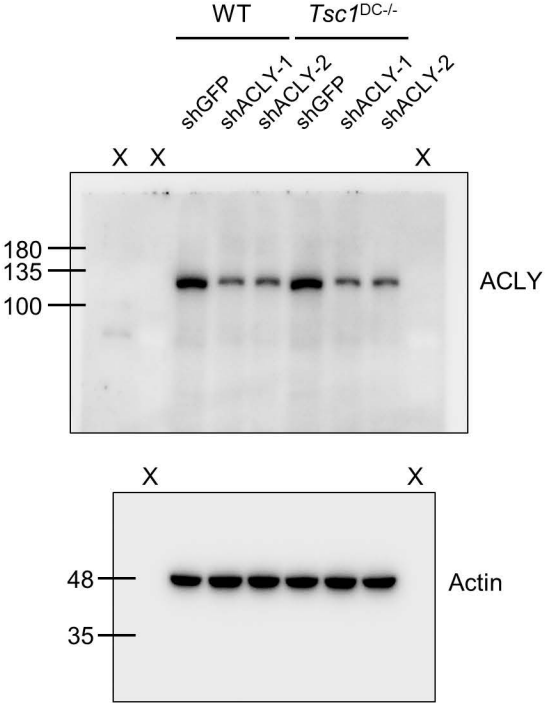

S1B Fig

cDCs

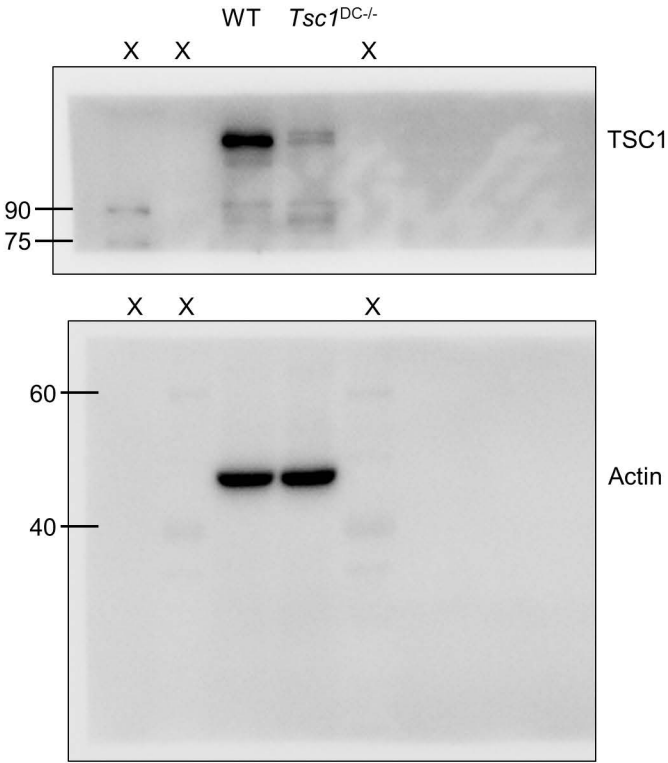

CD8 T cells

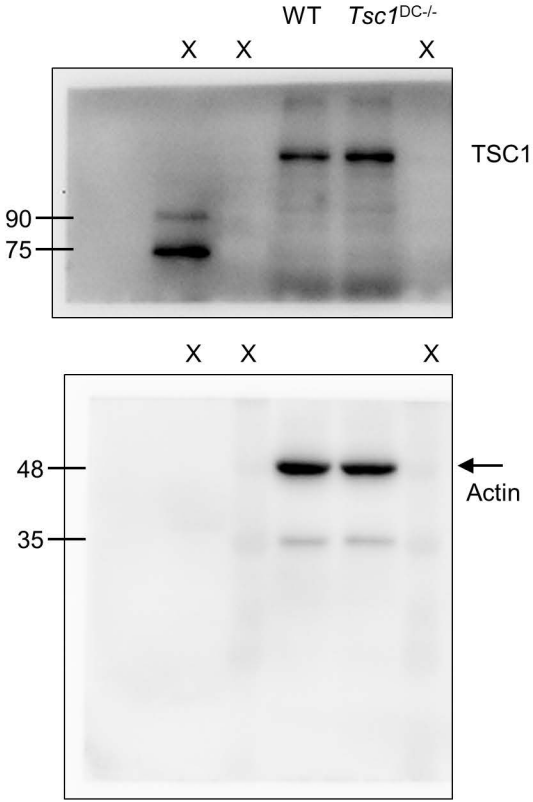

CD4 T cells

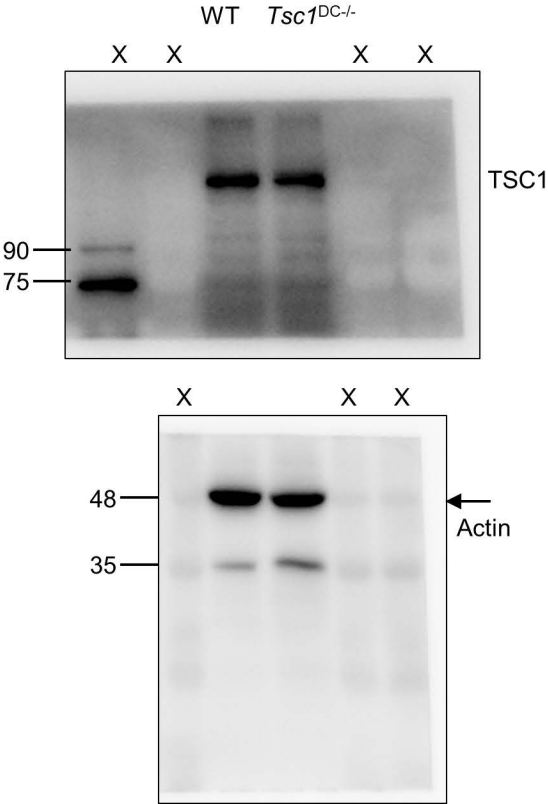

S7A Fig

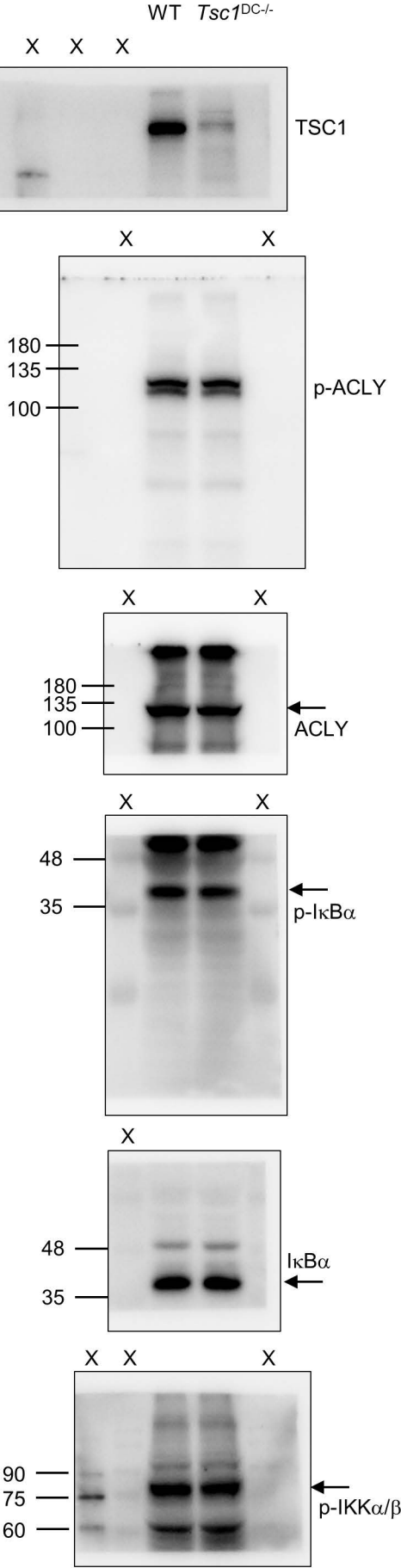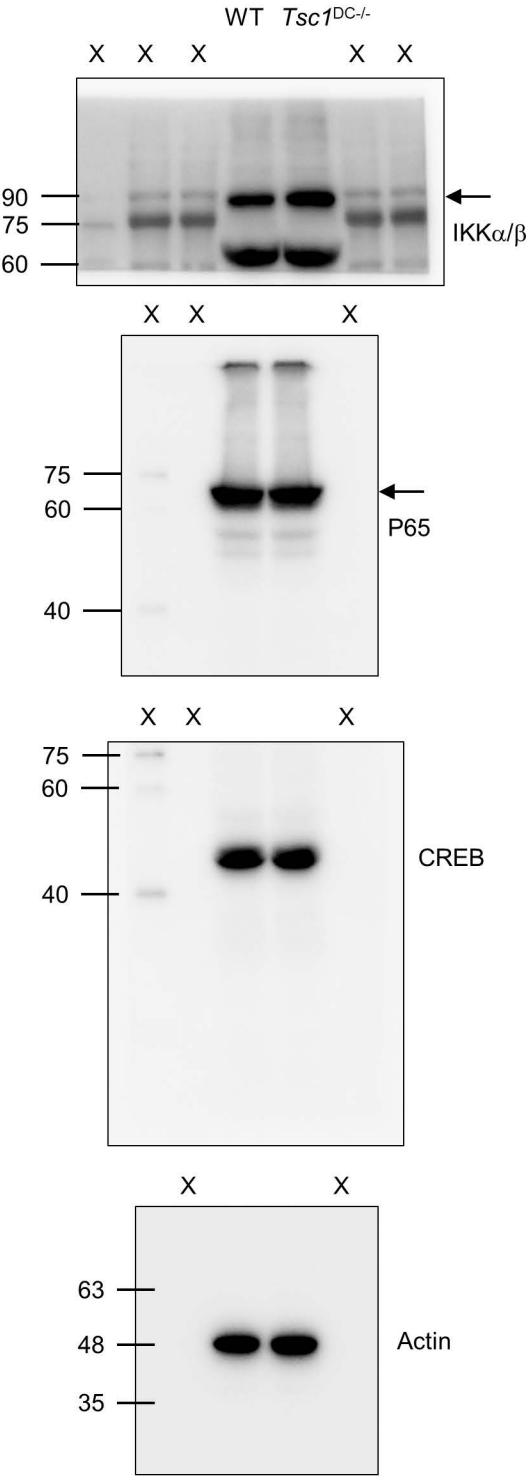

Supplement: S1 Raw Images — (PDF) [file pbio.3000420.s010.pdf]
